# Repetitive Transcranial Magnetic Stimulation Alters Brain Communication Networks to Improve the Cognitive Function in Patients with Amnestic Mild Cognitive Impairment - A Clinical Trial

**DOI:** 10.2174/011570159X389814251019160424

**Published:** 2026-02-12

**Authors:** Zhiyuan Yang, Moran Tangli, Yi Tan, Haojie Chen, Ni Shu, Zhihong Ke, Zheqi Hu, Qing Ye, Hailan Meng, Haifeng Chen, Yun Xu

**Affiliations:** 1 Department of Neurology, Nanjing Drum Tower Hospital, Affiliated Hospital of Medical School, Nanjing University, Nanjing, China;; 2 Nanjing Drum Tower Hospital Clinical College of Nanjing Medical University, Nanjing, China;; 3 Nanjing Drum Tower Hospital Clinical College of Traditional Chinese and Western Medicine, Nanjing University of Chinese Medicine, Nanjing, China;; 4 State Key Laboratory of Pharmaceutical Biotechnology and Institute of Translational Medicine for Brain Critical Diseases, Nanjing University, Nanjing, China;; 5 Jiangsu Key Laboratory for Molecular Medicine, Medical School of Nanjing University, Nanjing, China;; 6 Nanjing Neurology Clinical Medical Center, Nanjing, China;; 7 State Key Laboratory of Cognitive Neuroscience and Learning & IDG/McGovern Institute for Brain Research, Beijing Normal University, Beijing, China

**Keywords:** Amnestic mild cognitive impairment, Alzheimer’s disease, repetitive transcranial magnetic stimulation, diffusion tensor imaging, brain communication network, random forest models

## Abstract

**Introduction:**

Repetitive Transcranial Magnetic Stimulation (rTMS) is a non-invasive intervention that could effectively enhance the cognitive function in patients with amnestic mild cognitive impairment (aMCI). However, the mechanism and predictive biomarkers for therapeutic response remain poorly understood.

**Methods:**

Fifty-three aMCI patients underwent either neuro-navigated rTMS (n=28) or sham stimulation (n=25) targeting the left angular gyrus over four weeks (registered in 2021: ChiCTR2100050496). Multimodal MRI and comprehensive neuropsychological assessments were conducted pre- and post-intervention. Changes in brain communication networks and their correlation with cognitive improvements were analysed, with random forest models applied to predict treatment efficacy.

**Results:**

Episodic memory (*p<*0.001) and general cognitive function (*p<*0.05) of aMCI patients were significantly improved after intervention. Novel alterations in brain communications networks were identified in 5 sensorimotor areas, executive control regions, and emotion-cognition processing hubs. Communication alterations between the right precentral gyrus and right angular gyrus were positively correlated with the improvements in episodic memory (r=0.38, *p=*0.046), while the alterations between right precentral gyrus and right angular gyrus were negatively correlated with improvements in general cognitive function (MMSE, r=-0.44, *p=*0.019; MoCA, r=-0.43, *p=*0.024). Notably, the random forest model integrating communication network patterns with baseline demographic and neuropsychological data showed strong power in predicting rTMS effects.

**Discussion:**

These findings advance understanding of rTMS mechanisms by linking network plasticity to cognitive gains, addressing critical knowledge gaps.

**Conclusion:**

Neuro-navigated rTMS targeting the left angular gyrus may enhance cognitive function in aMCI patients by improving inter-brain regions communication. Baseline communication patterns hold promise as predictive biomarkers, facilitating personalized treatment strategies.

**Clinical Trial Registration Number:**

ChiCTR2100050496.

## 1 INTRODUCTION

Alzheimer’s disease (AD), characterized by the development of cognitive decline, is a progressive neurodegenerative disease and the most common type of dementia worldwide [1]. Amnestic Mild cognitive impairment (aMCI) due to AD is generally regarded as a pathologic precursor to AD and would progress to AD at a rate of approximately 15-20% per year [2, 3]. Early treatment for aMCI is the key to delaying cognitive decline and preventing dementia progression [4, 5]. Currently, the effectiveness of pharmaceutical treatments in slowing cognitive decline has not reached expectations, and nonpharmacological interventions have attracted increasing attention [6]. It has been reported that nonpharmacological interventions such as cognitive training and non-invasive brain stimulation can improve cognitive function in aMCI patients [7, 8].

Repetitive transcranial magnetic stimulation (rTMS) is a non-invasive intervention that can regulate brain excitability and activity through cortico-subcortical networks by intervening at specific targets [9, 10]. Extracellular plaques and intracellular tangles are characteristic pathological alterations in AD, and the presence of plaques and tangles in the hippocampus is strongly associated with cognitive decline. However, owing to the depth of stimulation of rTMS, we cannot directly stimulate the hippocampus. Therefore, we choose brain regions that are closely related to the hippocampus as stimulation targets instead of the hippocampus. Based on our previous research [11], we set the target at the left angular gyrus (MNI: -45, -67, 38), which is also an important part of the memory network. The left angular gyrus may be a potential new target for rTMS to improve cognitive function in AD spectrum patients, and the intervention mechanism involved is worthy of further exploration.

Depending on the different definitions of network nodes and research methods, complex brain networks can be divided into functional networks, white/gray matter networks and so on [12, 13]. With the refinement of the brain connectivity atlas and advances in research techniques, communication networks, based on the diffusion tensor imaging (DTI) data, provide a better framework to explain nervous system activities at multiple scales [14], from synaptic transmission between neurons [15] to whole-brain patterns of regional coactivation [16]. Communication networks provide a flexible way to model interactions between interconnected neural elements by abstracting biological neural signals, such as fractional anisotropy and fibre number, into network communication dynamics [17]. The communication network model can well describe the transsynaptic transmission mode during the progression of AD [18] and better explain cognitive and neural communication disruptions in AD patients than other network models can [19]. The therapeutic effects of rTMS are diffused through interbrain connections between brain regions, which is similar to the communication network model. The application of a communication network may help improve the intervention efficacy of rTMS [20] and further investigate the underlying mechanism. Like patients receiving pharmaceutical treatments, not everyone responds to rTMS treatment [21]. Therefore, it would be highly interesting to predict a patient's baseline response to rTMS to optimize a patient's nondrug treatment strategy.

To our knowledge, this is the first research to study alterations in the communication network of aMCI patients and their associations with improvements in cognitive function after rTMS intervention. The left angular gyrus was chosen as the intervention target, and the neural navigation system guaranteed the positioning accuracy. We further used machine learning methods to explore the factors that may affect the efficacy of rTMS at baseline for aMCI patients, laying a foundation for improving rTMS stimulation strategies and providing an effective efficacy prediction model.

## 2 MATERIALS AND METHODS

### 2.1 Participants

 The Ethics Committee of Nanjing Drum Tower Hospital approved this study. This study was prospectively registered in 2021 in the China Clinical Trial Registry (www.chictr.org.cn, registration number: ChiCTR2100050496). All the subjects signed informed consent before participating in the experiment. Sixty elderly subjects participated in this study. Participants were randomly assigned to the rTMS group or the sham stimulation group. Three patients were excluded because of loss of imaging data, and four patients were excluded because of excessive head movement (more than three degrees) during MRI scanning. The remaining fifty-three patients were eventually included in the analysis (the rTMS group*=*28, the sham group*=*25). In accordance with the CONSORT (Consolidated Standards of Reporting Trials) guidelines, the flow of participants throughout this study is detailed in Fig. (**1**). These subjects were all diagnosed with aMCI clinically. The specific diagnostic criteria are based on the recommendations of previous studies [22, 23], as described below: (1) memory complaint confirmed by the subject and/or an informant; (2) objective cognitive performance documented by an auditory verbal learning test-delayed recall (AVLT-DR) score below or equal to 1.5 SD of education- and age-adjusted norms; (3) clinical dementia rating (CDR) score = 0.5; and (4) not sufficient for dementia. The exclusion criteria included the following points: severe brain trauma, epilepsy, brain tumors, Parkinson's disease, severe anxiety and depression, or other neurological or psychiatric disorders that may lead to memory loss. People with any contraindication for MRI or poor image quality were also excluded.

### 2.2 Experimental Design and Neuro-navigated rTMS

The therapeutic target for this study was calculated based on another AD spectrum cohort in our team. Our group explored intervention targets through seed-based functional connectivity analysis using the left hippocampus as a seed point. The experimental results revealed that the AD spectrum population exhibited significant differences in the left angular gyrus (MNI: -45, -67, 38) [11]. The tolerance of the angular gyrus to high-frequency stimulation and the safety of such stimulation have been confirmed by previous literature [24, 25]. Therefore, we selected this brain region as the therapeutic target. Patients included in this study were not duplicates of those included in previous studies.

All patients underwent a complete medical history, neurological examination, a neuropsychiatric evaluation, brain MRI scanning, and an extensive neuropsychological assessment exploring all the cognitive domains at the first visit. All the participants received neuro-navigation rTMS stimulation of the angular gyrus for 4 weeks. rTMS was applied daily from Monday to Friday. Brain MRI scanning and neuropsychological measurements were performed once again after four weeks of rTMS treatment. Details of the study design are summarized in Fig. (**2**).

rTMS was delivered with a 70‐mm figure-eight coil *via* a magnetic stimulator (CCY‐IV model; YIRUIDE Inc., Wuhan, China). The coil used in the sham group can produce the same vibration and sound as the real coil, but does not produce the corresponding magnetic field. A total of 1600 stimulations lasted approximately 20 minutes, consisting of forty circulations, and each circulation consisted of 2 seconds delivered at 20 Hz spaced out and 28 s of no stimulation. These parameters associated with rTMS intervention were based on previous studies [26]. The stimulation target is defined as a sphere with a radius of 6 mm centered on MNI coordinates (MNI: -45, -67, 38). This target was individually transformed into each participant’s T1 space *via* an inverse matrix produced during T1 segmentation *via* TMStarget software. Each individual’s stimulation target was subsequently imported into a neuro-navigation system (Visor 2.0, Advanced Neuro Technologies, Enschede, the Netherlands). The coil was set on the angular gyrus tangentially to the skull during the treatment. The intensity of stimulation was set at 100% of the resting motor threshold (RMT). The RMT was defined as the lowest intensity producing MEPs of >50 μV in at least five out of 10 trials in the relaxed first dorsal interosseous (FDI) muscle of the right hand. The RMT was assessed at the optimal stimulation site to elicit MEPs in the right FDI, which was considered the motor spot. When RMT was evaluated, patients were informed that the threshold test assessed brain excitability and that the position of the coil during RMT was far from the stimulation target and would not affect subsequent stimulation.

### 2.3 Brain Imaging Acquisition and Preprocessing

All participants were examined on a Philips 3.0-T scanner (Philips Medical Systems). The specific parameters are as follows: (1) T1-weighted turbogradient echo sequence: TR = 9.8 ms, TE = 4.6 ms, FA = 8°, FOV = 250 × 250 mm^2^, number of slices = 192, acquisition matrix = 256 × 256, thickness = 1.0 mm (2) diffusion-weighted imaging sequence: the number of gradient directions = 32 (b = 1000 s/mm^2^) and one b0 image, TR = 9.154 ms, TE = 55 ms, acquisition matrix = 112 × 112, FOV = 224 × 224 mm^2^, thickness = 2.5 mm, and voxel size = 2 × 2 × 2.5 mm^3^ (3) FLAIR sequence: TR = 4.500 ms, TE = 333 ms, time interval (TI) = 1.600 ms, number of slices = 200, and voxel size = 0.95 × 0 Preprocessing was performed with a previously published pipeline [27]. The preprocessing of diffusion MRI (dMRI) data involved several steps, including denoising, head motion correction, and eddy current distortion correction. For the T1-weighted image, brain extraction was performed using FSL’s bet command, followed by tissue segmentation with the fast command. The white matter (WM) segmentation, obtained in T1 space, was transformed into diffusion space and used as a seed mask for tractography. BedpostX was then applied to the preprocessed dMRI data to model the diffusion parameters at each voxel, with three fibers modeled per voxel, a burn-in period of 3000, and a deconvolution model using stick representations. A more detailed description of the pipeline has been provided elsewhere [27].

### 2.4 Neuropsychological Measurement

To assess the intervention effects of rTMS, a standardized neuropsychological test protocol was employed. The details are as follows: (1) Global cognitive function: Mini-Mental State Examination (MMSE) and Montreal Cognitive Assessment Beijing (MoCA-BJ). (2) Episodic memory: Auditory Verbal Learning Test-Delayed Recall (AVLT-DR) scores and the Wechsler Memory Scale-Visual Reproduction-Delayed Recall (VR-DR). (3) Information processing speed: Trail Making Test-A (TMT-A) and the Stroop Color and Word Tests A and B (Stroop A and B). (4) Language function: Boston Naming Test and Category Verbal Fluency test. (5) Executive function: Digit Span Test-backwards, Trail Making Test-B (TMT-B) and Stroop Color and Word Tests C (Stroop C). (6) Visuospatial function: Clock Drawing Test and Visual Reproduction-copy test. (7) Mental condition: Hamilton Anxiety Scale (HAMA) and Hamilton Depression Scale (HAMD). The raw test scores were converted to Z scores, which were used to calculate the compound cognitive index. The TMT-A, B, Stroop A, B, C test was first inverted and then Z-transformed. To prevent repetition effects, different versions of the neuropsychological tests were used before and after treatment.

The raw test scores were converted to Z scores for analysis, and a compound cognitive index was calculated: episodic memory was calculated as the average of the AVLT-DR and VR-DR; information processing speed was the average of the TMT-A and Stroop A and B; language function was the average of the BNT and CVF; executive function was the average of the TMT-B and Stroop C; and visuospatial function was the average of the CDT and VR-C. To avoid repetition effects, alternate versions of the neuropsychological tests were employed before and after the intervention. The cognitive subdomains were integrated according to previously published methods [22, 28] in the same cohort.

### 2.5 Brain Network Construction and Analysis

Structural brain networks were constructed *via* a previously established pipeline [27]. The construction was performed with the application of BedpostX and Camino for modelling fibre orientations and conducting deterministic tractography. Fractional anisotropy (FA) and the fibre number (FN) were used to weight the connectivity matrices separately, resulting in FA and FN-weighted adjacency matrices. The automated anatomical labelling (AAL) atlas, comprising 90 regions, was employed to define network nodes (Table **S1** for detailed descriptions). For each participant, a communicability matrix was calculated using the brainGraph package [28]. Briefly, the communicability matrix G*_i,j_* measures the efficiency with which information is exchanged between pairs of nodes in the network, taking into account all possible paths (both direct and indirect) between them:


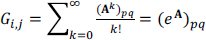


where *k* is the *walk* length.

### 2.6 Statistical Analysis

The power for the sample size test was calculated using G∗Power (V3, https://www.psychologie.hhu.de/). Baseline and after intervention demographic and clinical data were statistically analysed using SPSS software (V25, Chicago, IL, USA). Baseline comparisons between the rTMS group and the sham group were conducted by a 2-sample t-test. Differences in general cognitive function and multi-model neuropsychological test scores before and after intervention were analysed using a 2-way (group × time) repeated measures ANOVA test. Two-sided *p* < 0.05 was considered statistically significant.

For the analysis of structural changes in the communicability matrices, we adopted linear mixed-effect models. These models accounted for covariates such as age, sex, and years of education. To enhance precision, individualized mixed effects were tailored for each participant. Moreover, we employed network-based statistics (NBS) [29] with 10,000 permutations to ensure the robustness of the observed changes and to guard against false-positive results.

To investigate the relationship between communication network strength and specific cognitive test variations pre- and post-intervention, we calculated edgewise partial correlations within the mask of significant edges identified through NBS correction. We incorporated covariates such as age, sex, and educational background to control for potential confounding factors. To address multiple comparisons, false discovery rate (FDR) correction was employed across edges that are significant through NBS

### 2.7 Predicting Cognitive Function after Intervention based on Brain Communication

We adopted machine models to assess the effects of interventions on cognitive test scores *via* a Random Forest (RF) algorithm, tailored for a dataset with a limited sample size (N=28). The methodological pipeline commenced with rigorous data preprocessing, where continuous variables, including age, years of education, baseline cognitive test scores, and related edge-wise connection weights at baseline, were standardized to ensure equitable contributions to the model. To optimize the RF parameters, Leave-One-Out Cross-Validation (LOOCV) was utilized, maximizing data use by iteratively training the model on all but one sample and testing it on the excluded sample, thereby identifying the optimal number of candidate variables (mtry). Following parameter optimization, we evaluated the model's performance *via* the mean absolute error (MAE), root mean squared error (RMSE), and R-squared (R²) to obtain the predictive accuracy and variance explanation. Variable importance was elucidated through partial dependence plots, offering insights into the impact of each predictor on cognitive outcomes. To further evaluate the robustness and generalizability of our model, internal validation was conducted by bootstrapping involving 5,000 resamplings with replacement. The 95% confidence intervals of the MAE, RMSE, and R² were derived from the bootstrapped sample to evaluate the reliability of the model.

## 3 RESULTS

### 3.1 Baseline Demographic and Clinical Characteristics

Baseline demographic and clinical data in the rTMS group and sham group are shown in Table **1**. There were no significant baseline differences between the rTMS group and the sham group in any demographic information or cognitive-related variables.

### 3.2 Neuropsychological Tests Alteration after Intervention

Two-way repeated measures ANOVA was conducted to assess changes before and after intervention between the rTMS group and sham group (Table **2**). The analysis shown significant group × time interactions for MoCA (F= 13.938, *P <* 0.001), HAMA (F= 5.069, *P* = 0.029), HAMD (F= 13.338, *P* = 0.001), Episodic memory (F= 11.029, *P* = 0.002) including AVLT-DR(F= 7.015, *P* = 0.011) and VR-DR (F=6.421, *P* = 0.014), Language function (F= 10.115, *P* = 0.002) including BNT (F= 4.880, *P* = 0.032), Stoop C tests (F= 5.654, *P* = 0.021). A post reveal test revealed that patients receiving active rTMS showed better performance in MoCA (t= -3.877, *p=*0.001), HAMA (t=3.868, *P* = 0.001), HAMD (t=4.020, *P <* 0.001), Episodic memory (t=-5.766, *P <* 0.001) including AVLT-DR (t= -6.196, *P <* 0.001) and VR-DR (t=-3.249, *P* = 0.003 Language function (t= -4.213, *P <* 0.001) including BNT (t= -4.492, *P <* 0.001).

### 3.3 Intervention-related Communicability Changes

The intervention yielded significant alterations (NBS *P <* 0.05) in communicability connectivity patterns across critical brain regions (Fig. **3**) in rTMS group, including altered communication connectivity between the right precentral gyrus and the right supplementary motor area, regions involved in sensorimotor processing (Table **S2**). Altered brain communication between the right superior frontal gyrus, left medial superior frontal gyrus, and left inferior frontal gyrus (opercular part) was also observed, which are parts of executive control regions. Additionally, changes were observed in regions linked to emotion-cognition integration, particularly between the left orbitofrontal cortex and left anterior cingulate cortex. These findings suggest that rTMS may induce functional reorganization in motor, executive, and emotional processing circuits.

### 3.4 Communicability-cognition Relationships Following Intervention

Our analyses revealed significant relationships between changes in cognitive functions and alterations in brain communication (Fig. **4**).65 significant connections are remaining after FDR correction (Details shown in Table **S3**). Specifically, we observed a negative correlation between cognitive changes, particularly MoCA (R = −0.43, P = 0.024) and MMSE scores (R = −0.44, *p* = 0.019) (Fig. **4A**), and edgewise communicability alterations within prefrontal cortical regions, whereas memory functions exhibited strong connectivity with the hippocampus and temporal lobes. The altered communication ability between the left olfactory cortex and left superior parietal gyrus was positively correlated with improved episodic memory (R = 0.38, *P* = 0.046) (Fig. **4B**). In addition, a positive correlation was identified between changes in language function and edge-wise communicability between the left inferior occipital gyrus and the left precuneus (R = −0.40, *P* = 0.036) (Fig. **4C**). After controlling for baseline cognitive performance, the episodic memory was positively correlated with changes in brain communication between the left olfactory cortex and left superior parietal gyrus (r = 0.43, *p* = 0.023), as well as that for language function was negatively correlated with communication between right amygdala and right precuneus (r = -0.41, *p* = 0.031) (Fig. **4D**). These region-specific correlations suggest that the distinct neural architecture underlying improvements in different cognitive domains.

### 3.5 Random Forest Models Predicting Cognitive Performance Postintervention

The random forest models demonstrated strong predictive power in forecasting postintervention cognitive performance, integrating preintervention cognitive scores, network communication patterns, and demographic variables (Fig. **5**). Among these factors, preintervention cognitive status emerged as a critical determinant of future cognitive outcomes. The models exhibited robust predictive capacity for intervention-related improvements in language ability, information retrieval, episodic memory, and executive function (Fig. **S1** and Table **S4**). These results were rigorously validated through optimized model selection and extensive testing, including validation *via* 5000 bootstrap iterations, confirming the reliability and generalizability of the predictive framework.

## 4 DISCUSSION

Our study was the first to investigate alterations in the brain communication network in aMCI patients through 4 weeks of navigated rTMS intervention targeting the left angular gyrus. After rTMS intervention, the cognitive function of aMCI patients improved. In regard to the brain communication network, changes within key brain regions were the first time to be observed, including sensorimotor areas, executive control regions. The improvement of cognitive function was correlated with enhanced communication between the key brain regions. We further constructed a machine learning model to explore the factors influencing cognitive function after rTMS intervention. This study may contribute to a better understanding of how the cognitive function in aMCI patients is enhanced by improving the communication ability of key nodes in patients' brain regions through neuro-navigation rTMS.

### 4.1 Importance and Progress of rTMS Target Selection in aMCI Patients

How to effectively intervene in the early stage of Alzheimer's disease and delay its progression has always been an urgent problem to be solved [30, 31]. The choice of intervention target is the key factor for rTMS to play a therapeutic role. Traditional rTMS tends to intervene in classical anatomical locations such as the dorsolateral prefrontal cortex (DLPFC) and pre-cuneus [32]. Our research represents the first study to target the angular gyrus, which is based on the basis of characteristic brain changes in AD patients according to previous research by our team [11]. Compared with traditional targets, such as the DLPFC, the angular gyrus has its own advantages. The angular gyrus is a critical Default mode network (DMN) node, densely connected to the hippocampus and precuneus [16], which are central to episodic memory encoding and retrieval. In contrast, DLPFC primarily resides in the executive control network. AD-related tau propagation preferentially affects DN hubs like the angular gyrus [18], making it a strategic target to restore disrupted memory networks. Besides, DLPFC stimulation typically targets executive function, while angular gyrus stimulation directly modulates memory networks. This is reflected in our data: angular gyrus rTMS improved both global cognition (MMSE/MoCA) and specific memory domains (AVLT-DR, VR-DR), whereas prior DLPFC studies show more modest memory effects [20]. After rTMS intervention, episodic memory function language function, and overall cognitive function improved, and anxiety and depression were also relieved. These findings may provide new possibilities for how to effectively intervene early in AD.

Memory impairment is a major symptom in AD patients and the angular gyrus are largely involved in maintaining episodic memory function [33, 34]. The long-term potentiation model revealed that the angular gyrus plays an important role in the retrieval and recall of episodic memory [35]. The angular gyrus is also an important part of the language centre [36, 37], which may explain why language function is improved after rTMS. Inhibitory TMS targeting the left angular gyrus affects details related to internal (*i.e*., episodic) details in memory tasks while increasing the production of external details (*i.e*., repetitive or off-topic information) [38, 39]. rTMS stimulation targeting the angular gyrus has great potential for improving the memory function of AD patients.

### 4.2 Angular Gyrus

In addition to the core features of cognitive dysfunction, AD is accompanied by neuropsychiatric symptoms. According to a meta-analysis, approximately 30-40% of AD patients are complicated with varying degrees of depression and anxiety [40, 41], and rTMS has been confirmed to relieve depression or anxiety in patients. Notably, there is a correlation between the degree of depression and the degree of dementia [42]. rTMS can improve the sleep status and sleep of AD spectrum patients with insomnia by activating sleep-related networks [43]. Longitudinal studies have shown that anxiety severity in aMCI predicts accelerated conversion to AD, independent of depression and baseline cognitive deficits [44]. Specifically, anxiety symptoms in aMCI are associated with greater atrophy in the entorhinal cortex, a key region for memory processing, highlighting the neuroanatomical overlap between emotional and cognitive dysfunction [44]. While rTMS-mediated mood improvement likely provides an “environmental optimization” for cognitive performance by reducing anxiety-related distractibility and enhancing task engagement, the core mechanism of cognitive enhancement appears to involve structural and functional reorganization of brain networks. For example, rTMS targeting the DLPFC has been shown to normalize hyperactivity in the amygdala and strengthen connectivity between the DLPFC and hippocampus, thereby improving both emotional regulation and memory consolidation [45]. Furthermore, studies combining rTMS with neuroimaging have demonstrated rapid network reorganization in motor and language domains, suggesting that similar mechanisms may underlie cognitive improvements in aMCI [46]. These findings collectively support the notion that rTMS acts through network-level plasticity rather than merely alleviating emotional symptoms. The relationships among rTMS, neuropsychiatric symptoms and cognitive function may provide new ideas for the early prevention and treatment of AD.

### 4.3 The Underlying Mechanism of the Brain Communication Network Changes after rTMS Treatment

Multiple network alterations may be related to the propagation effects of rTMS as the stimulation is not limited to a certain point but has a spreading effect [26]. The excitatory/inhibitory effects of TMS can be transmitted from the stimulation target to distant brain regions through structural or functional circuits [47]. Therefore, the therapeutic effect of rTMS is often reflected in many aspects. A study focused on apathy in aMCI patients revealed that, in addition to changes in psychiatric symptoms, general cognitive function improved after rTMS intervention [48]. Another study revealed that rTMS intervention in episodic memory-related brain regions can improve both memory function and executive function [49], which is consistent with the results of our study. This may be related to the increasing therapeutic effect of rTMS. rTMS improves the ability to communicate information between multiple brain regions, which are not solely responsible for a certain function. As a result, we firstly studied the brain communication networks of aMCI patients after rTMS and found communication alterations in key brain regions, such as the precentral gyrus, superior frontal gyrus and anterior cingulate gyri, which play crucial roles in sensorimotor function, memory and emotional processing [41, 50].

We also found that the therapeutic effects of TMS in different cognitive domains were correlated with communication network changes. The communication network model describes a strategy for guiding signal propagation through structural connectivity. Common network features, such as efficiency and cost, can be quantified by communication model metrics, which provide a more intuitive perspective for understanding brain changes. In our study, we found that improvement in patients' episodic memory ability was positively correlated with changes in network communication in the superior parietal gyrus. An fMRI study reported that the superior parietal gyrus and olfactory cortex were activated in recognition memory tasks [51]. In people at high risk of cognitive impairment, activation in the superior parietal lobe is associated with reduced memory performance [52]. rTMS may enhance memory in aMCI patients by improving their ability to communicate with other brain regions of the superior parietal gyrus, which plays an important role in maintaining normal memory performance. Interestingly, the improvement in patients’ general cognitive function was negatively correlated with an altered communication network between the right precentral gyrus and right angular gyrus. This finding may reflect the optimization of network efficiency and regulation of frontal compensation after rTMS. The optimization of network efficiency can reduce network connectivity and the long-range pathways to enhance modular information processing [53]; And the regulation of frontal compensation, whereby decreased coupling may alleviate prefrontal cortex (PFC) hyperactivation, enables memory networks to operate independently. Baseline hyperconnectivity in this pathway likely reflects compensatory PFC over recruitment in aMCI, which aligns with the HAROLD mode [54] and frontal compensation theory [55]. The negative correlation thus suggests that rTMS-induced connectivity reduction optimizes network efficiency while reducing PFC compensatory load, facilitating cognitive restoration.. It has been reported that compensatory effects in the frontal lobe may protect cognitive function from the effects of aging in AD patients [56], which manifests as increased fALFF values and enhanced functional connections with other brain regions in the right precentral gyrus [57]. The network properties of the right precentral gyrus were revealed to be associated with cognitive reserve markers [58]. These findings suggest that frontal lobe-related brain areas, such as the precentral gyrus, have the ability to maintain cognitive function without impairment. Therefore, we hypothesized that after rTMS intervention, the therapeutic effect of rTMS may play a compensatory role and reduce the workload of frontal brain regions.

### 4.4 Factors Influencing the Therapeutic effect of rTMS

rTMS is considered to be a neuroregulatory technique that can effectively improve cognitive function in aMCI patients and delay their progression to AD, but the therapeutic effect of rTMS is not once for all. There are many factors that affect the efficacy of rTMS, such as hormone levels [59], family intervention [60], and the location of intervention targets [61, 62]. Traditional localization methods of rTMS generally rely on body surface landmarks [48] or brain zoning systems such as the EEG 10-20 system [63] for localization. These localization methods have the advantages of simple operation but lack accuracy. It is not enough to consider the overall size and shape of the head when determining an intervention target because the different cortical morphologies and connectivity vary greatly from person to person. To improve the rTMS treatment effect, it is necessary to improve the localization accuracy of the probe on the scalp to ensure that the focal magnetic field accurately intervenes in the target brain region, which is the advantage of neural navigation systems. The neural navigation system greatly improves the accuracy of localization, ensuring that researchers can accurately intervene in the target brain region [64]. More accurate auxiliary positioning methods, such as assistant robots and artificial intelligence positioning systems, will improve the therapeutic effect in the future. It is worth noting that, in addition to short-term therapeutic effects, long-term therapeutic effects are also of concern to people. It has been reported that the patient's cognitive function is partially maintained at 6 months after treatment [65]. However, the evolution of aMCI is a long-term process, so it is necessary to further follow up on the efficacy of rTMS. In the long-term effect study, we will conduct follow-up at multiple time points, including 3, 6, 12, 18, 24 and 36 months and so on after rTMS treatment, to better understand the long-term efficacy of TMS in the treatment of aMCI.

In this study, we constructed a machine learning prediction model using network communication patterns, patient demographic data and baseline cognitive function. The prediction model can accurately predict the intervention ability of rTMS in different cognitive domains, among which the cognitive function at baseline had a great influence on the prediction accuracy. The less impaired the cognitive function of aMCI patients is, the better the therapeutic effect of rTMS, which is consistent with the philosophy of early treatment in AD patients [66]. When AD progresses to the advanced stage, irreversible damage, such as white matter fibre tract breakage and hippocampus atrophy, occurs in the patient's brain [67, 68]. Therefore, it is more difficult for rTMS to exert therapeutic effects through network communication between brain regions [69]. Compared with aMCI patients with worse cognition, aMCI patients with better cognition have less brain damage [70], which provides a structural basis for rTMS exerting therapeutic effects. In addition, better cognitive function may be related to better cognitive reserve in related brain regions [71]. The cognitive reserve can increase tolerance to pathology in the brain while allowing some people to adapt to changes in the brain better than others do [72]. Some special TMS stimulation paradigms may enhance cognitive reserve and improve cognitive function [73]. To enhance clinical applicability, we propose patient selection criteria integrating baseline cognitive metrics (MoCA ≥22, MMSE ≥26, AVLT-DR ≤1.5 SD below age-normed scores), brain communication biomarkers (left olfactory cortex–superior parietal gyrus connectivity ≥ median, right precentral gyrus–angular gyrus connectivity ≤ median), and demographic factors (age ≤75, HAMA/HAMD <14/17). These criteria may contribute to the effective selection of populations for rTMS intervention in the clinic. However, there is still a lack of research on the correlation between cognitive reserve and rTMS. The specific mechanism of the synergistic effect between cognitive reserve and rTMS deserves further study.

## 5 LIMITATIONS

There are several limitations in our study. First, our complex design results in a relatively small number of patients. A small sample size may lead to overfitting of the machine learning model, and it is necessary to conduct multi-center and large sample prospective studies. Second, owing to time constraints, we did not study long-term effects in this study, and we intend to follow these patients over a long period of time. Hence, our study focused only on the short-term effects of rTMS treatment, awaiting further investigations of long-term effects. We lack a blinded assessment questionnaire to verify the effectiveness of blinding, and follow-up related studies are necessary. Finally, compared with traditional methods of network construction, communication networks are more interpretable, but the physiological significance of communication networks still needs further study [74].

## CONCLUSION

Our study revealed that after 4 weeks of rTMS intervention targeting the angular gyrus, the MCI group showed varying degrees of improvement in multidomain cognitive function. In addition, inter-regional brain communication of patients improved, which was correlated with improvements in cognitive function. The random forest model can effectively predict rTMS efficacy, in which cognition at baseline plays an important role in predicting subsequent cognitive outcomes. The identification of baseline communication patterns as predictors of treatment response provides novel insights for developing personalized neuromodulation strategies in prodromal Alzheimer's disease populations.

## Figures and Tables

**Fig. (1):**
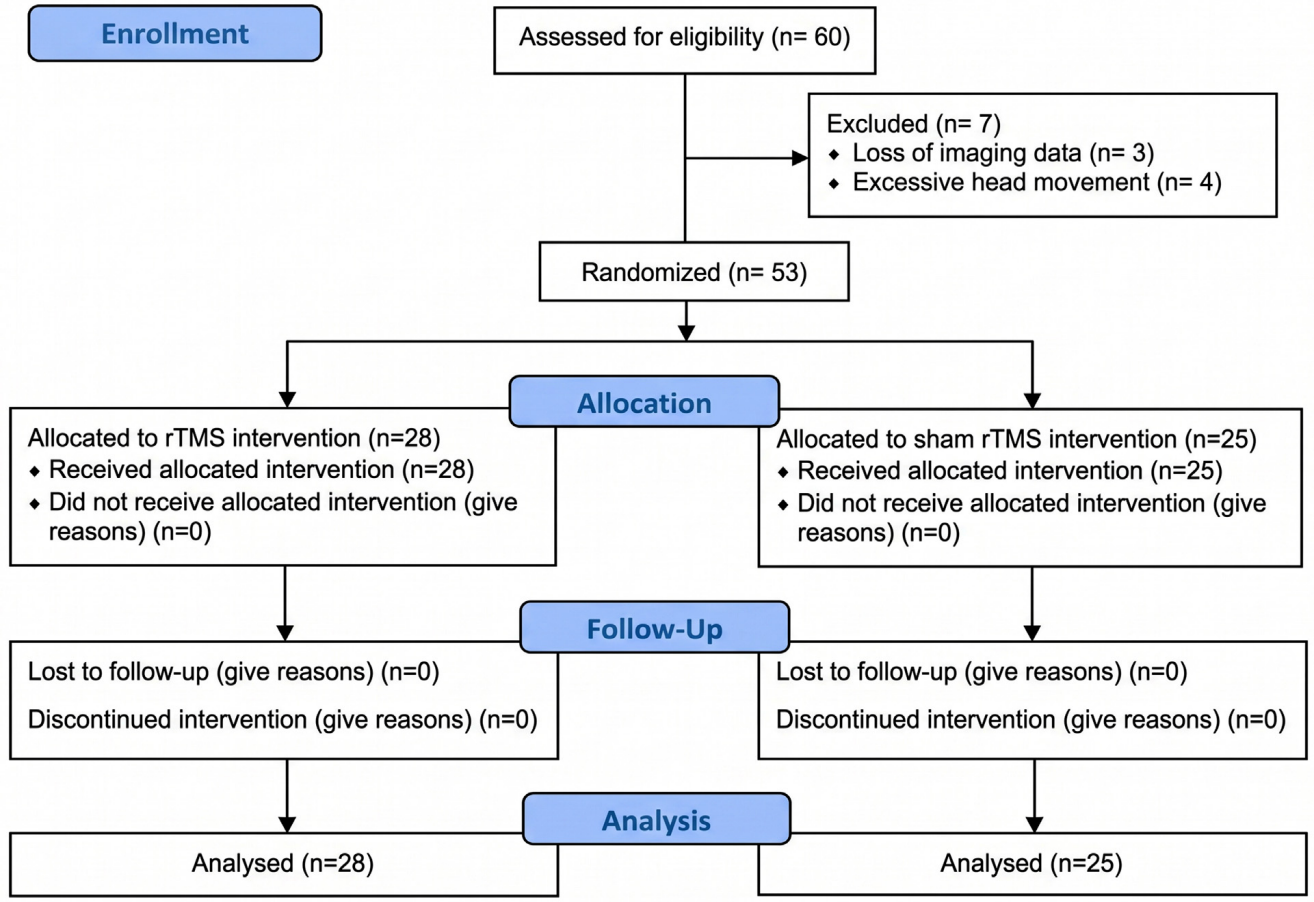
CONSORT Flowchart of Participant Flow. The specific process includes enrollment, allocation, follow-up, and analysis.

**Fig. (2):**
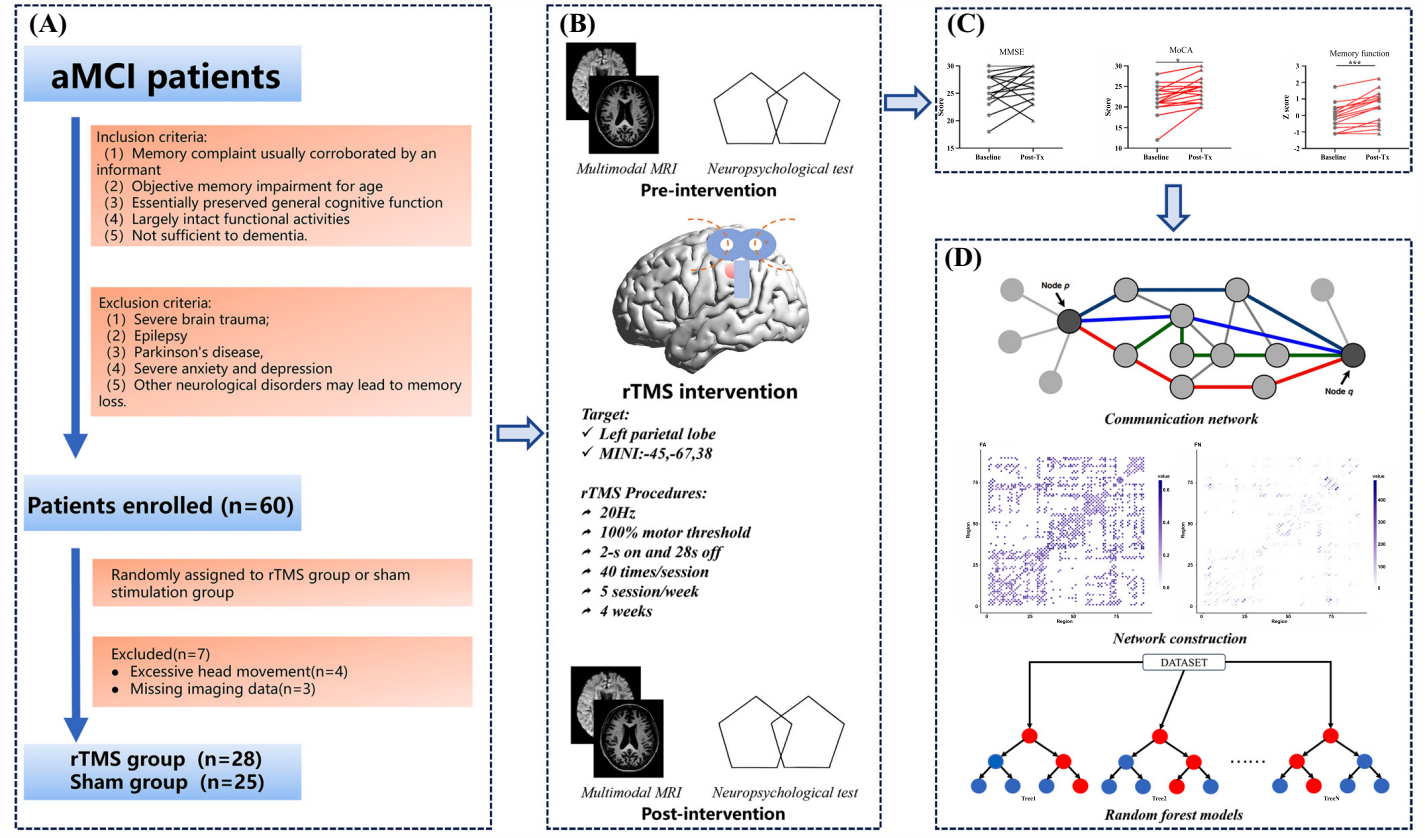
Study and experimental design. (**A**) The inclusion and exclusion criteria of MCI patients. (**B**) The experiment protocol and specific parameters of rTMS intervention. (**C**) Compare the changes of cognitive function in multiple cognitive domains. (**D**) Construct communication network and related random forest model based on MRI data. MCI, mild cognitive impairment; rTMS, repetitive transcranial magnetic stimulation; MRI, magnetic resonance imaging.

**Fig. (3):**
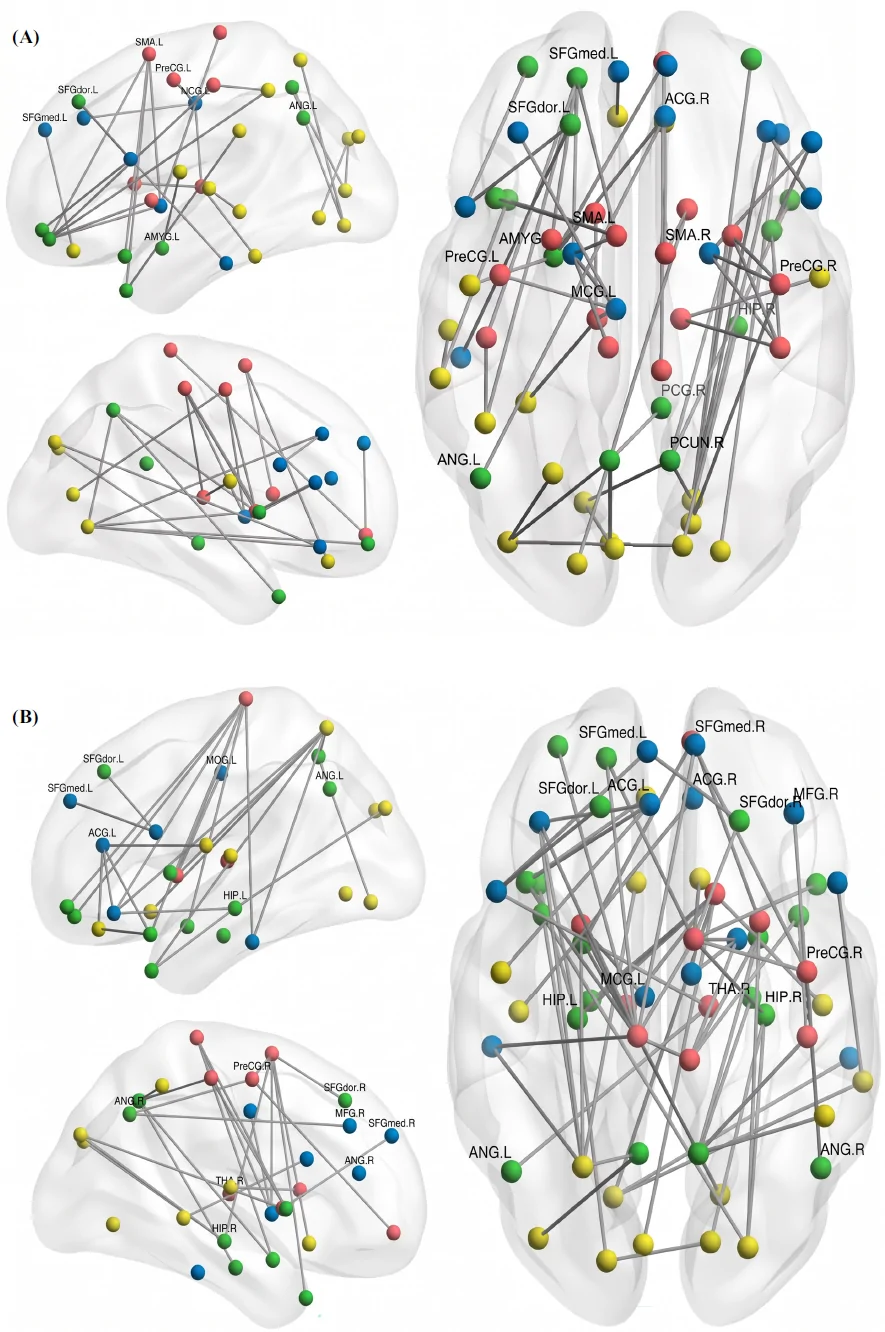
Changes in Edge-Wise Network Communicability Following TMS Intervention. Significant clusters (NBS-corrected, *P <* 0.05) were observed, primarily centered in sensorimotor areas, executive control regions, and emotion-cognition processing hubs in both the FA-weighted (**A**) and the FN-weighted (**B**) networks. Red nodes represent hubs in sensorimotor areas, blue nodes represent hubs in executive regions and green nodes represent hubs in emotion-cognition network. NBS, network-based statistics; FA, fractional anisotropy; FN, fibre number.

**Fig. (4):**
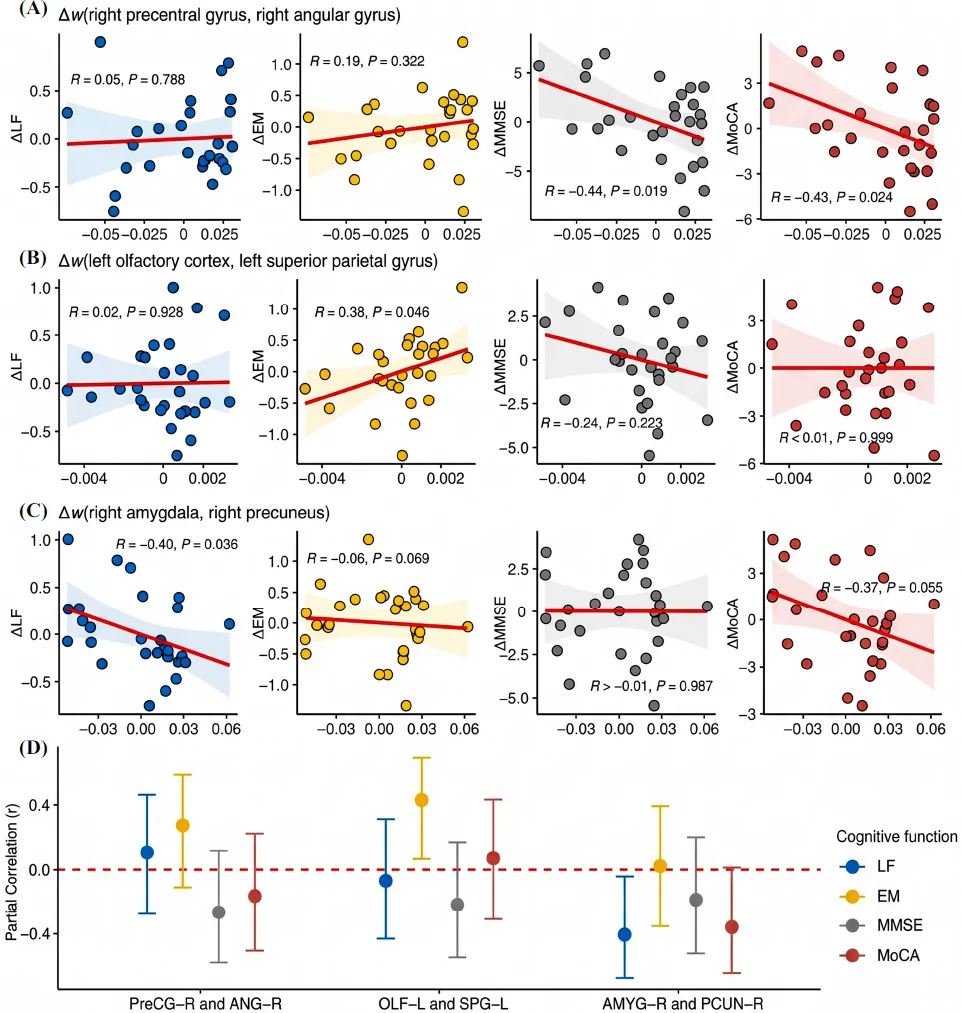
Brain-cognition correlations Correlations between changes in edge-wise brain communication and cognitive function. (**A**) The right precentral gyrus and right angular gyrus was negatively correlated with improvements in MMSE (R = −0.44, *P* = 0.019) and MoCA (R = −0.43, *P* = 0.024) scores. (**B**) The altered communication ability between the left olfactory cortex and left superior parietal gyrus was positively correlated with improved episodic memory (R = 0.38, *P* = 0.046). (**C**) The altered communication ability between the right amygdala and right precuneus was negatively correlated with changes in language function (R = −0.40, *P* = 0.036). (**D**) After controlling for baseline cognitive performance, the episodic memory was positively correlated with changes in brain communication between the left olfactory cortex and left superior parietal gyrus (r = 0.43, *p* = 0.023), as well as that for language function was negatively correlated with communication between right amygdala and right precuneus. X-axis represents the alteration of inter-brain communication ability after intervention and the Y-axis represents the alteration of cognitive function after intervention. LF, language function. EM, episodic memory. MMSE, Mini-Mental State Examination; MoCA, Montreal Cognitive Assessment.

**Fig. (5):**
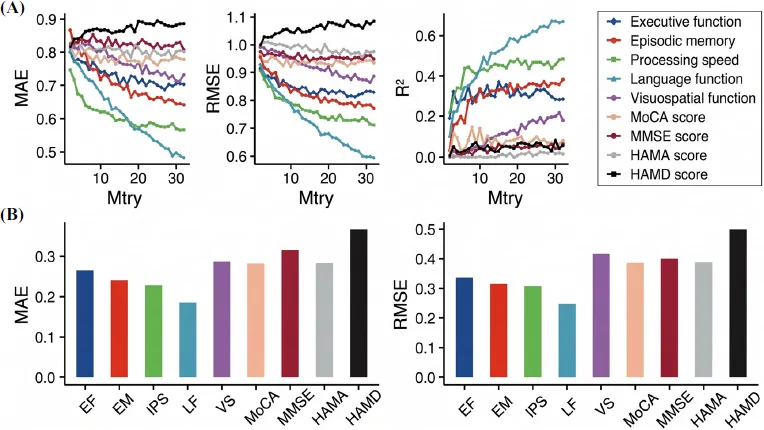
Random Forest (**A**) As Mrty increased, the prediction accuracy gradually increased, especially the change in episodic memory. (**B**) Model validation with 5000 bootstrap replicates. Mtry, the optimal number of candidate variables. EF, executive function. LF, language function. EM, episodic memory. IPS, information processing speed. VS, visuospatial function. MMSE, Mini-Mental State Examination; MoCA, Montreal Cognitive Assessment. HAMD, Hamilton Depression Scale. HAMA, Hamilton Anxiety Scale. MAE, mean absolute error. RMSE, root mean squared error.

**Table 1:** Baseline demographic and clinical characteristics of rTMS and sham groups.

**Characteristic**	**rTMS Group (N=28^1^)**	**Sham Group (N=25^1^)**	***p*-value** ^2^
**Demographic Characteristics**			
Age	66.89(1.33)	66.08 (1.58)	-
Sex	-	-	-
Female	16 (57%)	14(56%)	-
Male	12 (43%)	11(44%)	-
Education	12.17(0.54)	12.27(0.72)	-
**Baseline Clinical Characteristics**			
MMSE	26.86(0.47)	27.54(0.53)	0.342
MoCA	22.71(0.58)	23.62 (0.84)	0.376
HAMA	9.00(0.88)	7.23(0.85)	0.230
HAMD	6.00(1.13)	4.38(0.24)	0.088
Episodic memory	-0.38(0.15)	-0.02(0.16)	0.109
AVLT-DR	-0.43(0.17)	-0.11(0.18)	0.194
VR-DR	-0.33(0.16)	0.07(0.21)	0.137
Information processing speed	-0.13(0.13)	-0.03(0.19)	0.672
TMT-A	-0.27(0.00)	0.16(0.02)	0.135
Stroop A	0.00(0.00)	-0.24(0.04)	0.381
Stroop B	-0.12(0.01)	-0.02(0.04)	0.706
Language function	-0.10(0.16)	-0.14(0.14)	0.569
BNT	-0.18(0.19)	-0.06(0.19)	0.681
CVF	0.09(0.20)	-0.28(0.18)	0.178
Executive function	-0.13(0.13)	-0.07(0.16)	0.774
TMT-B	-0.25(0.00)	0.03(0.01)	0.311
Stroop C	-0.01(0.00)	-0.24(0.03)	0.382
Visuospatial function	-0.04(0.18)	-0.13(0.24)	0.773
CDT	0.04(0.18)	-0.10(0.22)	0.626
VR-C	-0.13(0.21)	-0.16(0.28)	0.925

**Abbreviations**: MMSE, Mini-Mental State Examination; MoCA, the Montreal Cognitive Assessment., HAMD, Hamilton Depression Scale. HAMA, Hamilton Anxiety Scale; AVLT-DR, auditory verbal learning test-delayed recall; VR-DR, Visual Reproduction-Delayed Recall; TMT-A, TMT-B, Trail Making Test-A, B; Stroop A, Stroop B, Stroop C, Stroop Color and Word Tests A, B, C; BNT, Boston Naming Test; CVF, Category Verbal Fluency test; CDT, Clock Drawing Test; VR-C Visual Reproduction–copy test. rTMS, repetitive transcranial magnetic stimulation.

^1^Values are presented as the mean (standard error mean, SEM).

^2^Calculated as 2-sample t test.

**Table 2:** Group × time interaction effect analysis of clinical characteristics after intervention.

**Clinical Characteristics**	**Change in Clinical Characteristics after Intervention^1^**		**Interaction Effect (Group × Time)**	
	**rTMS Group (N=28^2^)**	**Sham Group (N=25^2^)**	**F**	***p*-value^3^**
MMSE	-1.0(0.39)	-0.69(0.41)	0.294	0.590
MoCA	-1.86(0.51)	0.89(0.53)	13.938	<0.001***
HAMA	4.04(0.95)	7.12(0.99)	5.069	0.029*
HAMD	2.96(0.56)	0.04(0.58)	13.338	0.001**
Episodic memory	-0.62(0.09)	-0.18(0.10)	11.029	0.002**
AVLT-DR	-0.79(0.13)	-0.29(0.14)	7.015	0.011*
VR-DR	-0.46(0.11)	-0.07(0.11)	6.421	0.014*
Information processing speed	-0.22(0.10)	-0.11(0.11)	0.655	0.422
TMT-A	-0.00(0.00)	0.00(0.00)	2.168	0.147
Stroop A	-0.00(0.00)	-0.00(0.00)	0.217	0.643
Stroop B	-0.12(0.92)	-0.02(1.09)	0.939	0.337
Language function	-0.38(0.08)	-0.03(0.08)	10.115	0.002**
BNT	-0.39(0.09)	-0.11(0.09)	4.880	0.032*
CVF	-0.10(0.13)	-0.18(0.14)	0.175	0.678
Executive function	-0.15(0.10)	-0.34(0.10)	1.910	0.173
TMT-B	0.01 (0.00)	0.01(0.00)	1.170	0.284
Stroop C	0.00(0.00)	-0.01(0.00)	5.654	0.021*
Visuospatial function	0.09(0.30)	-0.02(0.20)	0.179	0.674
CDT	0.00(0.19)	-0.10(0.19)	0.130	0.720
VR-C	-0.24(0.22)	-0.36(0.23)	0.147	0.703

**Abbreviations**: MMSE, Mini-Mental State Examination; MoCA, the Montreal Cognitive Assessment., HAMD, Hamilton Depression Scale. HAMA, Hamilton Anxiety Scale; AVLT-DR, auditory verbal learning test-delayed recall; VR-DR, Visual Reproduction-Delayed Recall; TMT-A, TMT-B, Trail Making Test-A, B; Stroop A, Stroop B, Stroop C, Stroop Color and Word Tests A, B, C; BNT, Boston Naming Test; CVF, Category Verbal Fluency test; CDT, Clock Drawing Test; VR-C Visual Reproduction–copy test. rTMS, repetitive transcranial magnetic stimulation.

^1^Changes in clinical characteristics were calculated based on the estimated marginal mean (Pre-stimulation minus post-stimulation).

^2^Values are presented as the mean (standard error mean, SEM).

^3^Calculated by repeated measures ANOVA.

∗Indicates a significance between groups. *p < 0.05, **p < 0.01, ***p< 0.001.

## Data Availability

All data generated or analyzed during this study are included in this published article.

## LIST OF ABBREVIATIONS

AAL: Automated Anatomical Labelling
AD: Alzheimer’s Disease
aMCI: Amnestic Mild Cognitive Impairment
AVLT-DR: Auditory Verbal Learning Test-delayed Recall
BNT: Boston Naming Test
CDT: Clock Drawing Test
CONSORT: Consolidated Standards of Reporting Trials
CVF: Category Verbal Fluency Test
DLPFC: Dorsolateral Prefrontal Cortex
DMN: Default Mode Network
dMRI: Diffusion MRI
EF: Executive Function
EM: Episodic Memory
FA: Fractional Anisotropy
FDI: First Dorsal Interosseous
FDR: False Discovery Rate
FN: Fibre Number
HAMA: Hamilton Anxiety Scale
HAMD: Hamilton Depression Scale
IPS: Information Processing Speed
LF: Language Function
LOOCV: Leave-One-Out Cross-Validation
MAE: Mean Absolute Error
MEPs: Motor Evoked Potentials
MMSE: Mini-Mental State Examination
MoCA: Montreal Cognitive Assessment
MoCA-BJ: Montreal Cognitive Assessment Beijing
MRI: Magnetic Resonance Imaging
NBS: Network-Based Statistics
R2: R-squared
RF: Random Forest
RMSE: Root Mean Squared Error
RMT: Resting Motor Threshold
rTMS: Repetitive Transcranial Magnetic Stimulation
Stroop A: Stroop Color and Word Test A
Stroop B: Stroop Color and Word Test B
Stroop C: Stroop Color and Word Test C
TMT-A: Trail Making Test-A
TMT-B: Trail Making Test-B
VR-C: Visual Reproduction-copy test
VR-DR: Visual Reproduction-Delayed Recall
VS: Visuospatial Function
WM: White Matter

## STANDARDS OF REPORTING

CONSORT guidelines and methodology were followed.
