# Supplementary material for: Repetitive Transcranial Magnetic Stimulation Alters Brain Communication Networks to Improve the Cognitive Function in Patients with Amnestic Mild Cognitive Impairment - A Clinical Trial
Source: Curr Neuropharmacol. 2026 Feb 12;24(11):1683–99. doi: 10.2174/011570159X389814251019160424 (PMC13647718; doi:10.2174/011570159X389814251019160424)
Supplement: Supplementary file 1 [file CN-24-11-1683_SD1.pdf]

Supplementary Material

Repetitive Transcranial Magnetic Stimulation Alters Brain Communication Networks to Improve the Cognitive Function in Patients with Amnestic Mild Cognitive Impairment - A Clinical Trial

Zhiyuan Yang[1,4,5,6], Moran Tangli[1], Yi Tan[1], Haojie Chen[7], Ni Shu[7], Zhihong Ke[2], Zheqi Hu[1], Qing Ye[1,4,5,6], Hailan Meng[1,4,5,6], Haifeng Chen[3,4,5,6] and Yun Xu[1,4,5,6,*]

[1]Department of Neurology, Nanjing Drum Tower Hospital, Affiliated Hospital of Medical School, Nanjing University, Nanjing, China; [2]Nanjing Drum Tower Hospital Clinical College of Nanjing Medical University, Nanjing, China; [3]Nanjing Drum Tower Hospital Clinical College of Traditional Chinese and Western Medicine, Nanjing University of Chinese Medicine, Nanjing, China; [4]State Key Laboratory of Pharmaceutical Biotechnology and Institute of Translational Medicine for Brain Critical Diseases, Nanjing University, Nanjing, China; [5]Jiangsu Key Laboratory for Molecular Medicine, Medical School of Nanjing University, Nanjing, China; [6]Nanjing Neurology Clinical Medical Center, Nanjing, China; [7]State Key Laboratory of Cognitive Neuroscience and Learning & IDG/McGovern Institute for Brain Research, Beijing Normal University, Beijing, China

Table S1. Definition of brain regions and coordinates of the automated anatomical labelling (AAL) 90 atlas.

Region	Full Name	Acronym	X	Y	Z
1	Precentral_L	PreCG-L	-38.65	-5.68	50.94
2	Precentral_R	PreCG-R	41.37	-8.21	52.09
3	Frontal_Sup_L	SFGdor-L	-18.45	34.81	42.2
4	Frontal_Sup_R	SFGdor-R	21.9	31.12	43.82
5	Frontal_Sup_Orb_L	ORBsup-L	-16.56	47.32	-13.31
6	Frontal_Sup_Orb_R	ORBsup-R	18.49	48.1	-14.02
7	Frontal_Mid_L	MFG-L	-33.43	32.73	35.46
8	Frontal_Mid_R	MFG-R	37.59	33.06	34.04
9	Frontal_Mid_Orb_L	ORBmid-L	-30.65	50.43	-9.62
10	Frontal_Mid_Orb_R	ORBmid-R	33.18	52.59	-10.73
11	Frontal_Inf_Oper_L	IFGoperc-L	-48.43	12.73	19.02
12	Frontal_Inf_Oper_R	IFGoperc-R	50.2	14.98	21.41
13	Frontal_Inf_Tri_L	IFGtriang-L	-45.58	29.91	13.99
14	Frontal_Inf_Tri_R	IFGtriang-R	50.33	30.16	14.17
15	Frontal_Inf_Orb_L	ORBinf-L	-35.98	30.71	-12.11
16	Frontal_Inf_Orb_R	ORBinf-R	41.22	32.23	-11.91
17	Rolandic_Oper_L	ROL-L	-47.16	-8.48	13.95
18	Rolandic_Oper_R	ROL-R	52.65	-6.25	14.63

1875-6190/26 $65.00+.00 © 2026 Bentham Science Publishers

Region	Full Name	Acronym	X	Y	Z
19	Supp_Motor_Area_L	SMA-L	-5.32	4.85	61.38
20	Supp_Motor_Area_R	SMA-R	8.62	0.17	61.85
21	Olfactory_L	OLF-L	-8.06	15.05	-11.46
22	Olfactory_R	OLF-R	10.43	15.91	-11.26
23	Frontal_Sup_Medial_L	SFGmed-L	-4.8	49.17	30.89
24	Frontal_Sup_Medial_R	SFGmed-R	9.1	50.84	30.22
25	Frontal_Med_Orb_L	ORBmed-L	-5.17	54.06	-7.4
26	Frontal_Med_Orb_R	ORBmed-R	8.16	51.67	-7.13
27	Rectus_L	REC-L	-5.08	37.07	-18.14
28	Rectus_R	REC-R	8.35	35.64	-18.04
29	Insula_L	INS-L	-35.13	6.65	3.44
30	Insula_R	INS-R	39.02	6.25	2.08
31	Cingulum_Ant_L	ACG-L	-4.04	35.4	13.95
32	Cingulum_Ant_R	ACG-R	8.46	37.01	15.84
33	Cingulum_Mid_L	MCG-L	-5.48	-14.92	41.57
34	Cingulum_Mid_R	MCG-R	8.02	-8.83	39.79
35	Cingulum_Post_L	PCG-L	-4.85	-42.92	24.67
36	Cingulum_Post_R	PCG-R	7.44	-41.81	21.87
37	Hippocampus_L	HIP-L	-25.03	-20.74	-10.13
38	Hippocampus_R	HIP-R	29.23	-19.78	-10.33
39	ParaHippocampal_L	PHG-L	-21.17	-15.95	-20.7
40	ParaHippocampal_R	PHG-R	25.38	-15.15	-20.47
41	Amygdala_L	AMYG-L	-23.27	-0.67	-17.14
42	Amygdala_R	AMYG-R	27.32	0.64	-17.5
43	Calcarine_L	CAL-L	-7.14	-78.67	6.44
44	Calcarine_R	CAL-R	15.99	-73.15	9.4
45	Cuneus_L	CUN-L	-5.93	-80.13	27.22
46	Cuneus_R	CUN-R	13.51	-79.36	28.23
47	Lingual_L	LING-L	-14.62	-67.56	-4.63
48	Lingual_R	LING-R	16.29	-66.93	-3.87
49	Occipital_Sup_L	SOG-L	-16.54	-84.26	28.17
50	Occipital_Sup_R	SOG-R	24.29	-80.85	30.59
51	Occipital_Mid_L	MOG-L	-32.39	-80.73	16.11
52	Occipital_Mid_R	MOG-R	37.39	-79.7	19.42

Region	Full Name	Acronym	X	Y	Z
53	Occipital_Inf_L	IOG-L	-36.36	-78.29	-7.84
54	Occipital_Inf_R	IOG-R	38.16	-81.99	-7.61
55	Fusiform_L	FFG-L	-31.16	-40.3	-20.23
56	Fusiform_R	FFG-R	33.97	-39.1	-20.18
57	Postcentral_L	PoCG-L	-42.46	-22.63	48.92
58	Postcentral_R	PoCG-R	41.43	-25.49	52.55
59	Parietal_Sup_L	SPG-L	-23.45	-59.56	58.96
60	Parietal_Sup_R	SPG-R	26.11	-59.18	62.06
61	Parietal_Inf_L	IPL-L	-42.8	-45.82	46.74
62	Parietal_Inf_R	IPL-R	46.46	-46.29	49.54
63	SupraMarginal_L	SMG-L	-55.79	-33.64	30.45
64	SupraMarginal_R	SMG-R	57.61	-31.5	34.48
65	Angular_L	ANG-L	-44.14	-60.82	35.59
66	Angular_R	ANG-R	45.51	-59.98	38.63
67	Precuneus_L	PCUN-L	-7.24	-56.07	48.01
68	Precuneus_R	PCUN-R	9.98	-56.05	43.77
69	Paracentral_Lobule_L	PCL-L	-7.63	-25.36	70.07
70	Paracentral_Lobule_R	PCL-R	7.48	-31.59	68.09
71	Caudate_L	CAU-L	-11.46	11	9.24
72	Caudate_R	CAU-R	14.84	12.07	9.42
73	Putamen_L	PUT-L	-23.91	3.86	2.4
74	Putamen_R	PUT-R	27.78	4.91	2.46
75	Pallidum_L	PAL-L	-17.75	-0.03	0.21
76	Pallidum_R	PAL-R	21.2	0.18	0.23
77	Thalamus_L	THA-L	-10.85	-17.56	7.98
78	Thalamus_R	THA-R	13	-17.55	8.09
79	Heschl_L	HES-L	-41.99	-18.88	9.98
80	Heschl_R	HES-R	45.86	-17.15	10.41
81	Temporal_Sup_L	STG-L	-53.16	-20.68	7.13
82	Temporal_Sup_R	STG-R	58.15	-21.78	6.8
83	Temporal_Pole_Sup_L	TPOsup-L	-39.88	15.14	-20.18
84	Temporal_Pole_Sup_R	TPOsup-R	48.25	14.75	-16.86
85	Temporal_Mid_L	MTG-L	-55.52	-33.8	-2.2
86	Temporal_Mid_R	MTG-R	57.47	-37.23	-1.47

Region	Full Name	Acronym	X	Y	Z
87	Temporal_Pole_Mid_L	TPOmid-L	-36.32	14.59	-34.08
88	Temporal_Pole_Mid_R	TPOmid-R	44.22	14.55	-32.23
89	Temporal_Inf_L	ITG-L	-49.77	-28.05	-23.17
90	Temporal_Inf_R	ITG-R	53.69	-31.07	-22.32

Table S2. Edge-wise altered brain communication after rTMS stimulation.

Region1	Region2	Weight
Frontal_Sup_L	Frontal_Inf_Oper_L	FA
Precentral_R	Supp_Motor_Area_R	FA
Frontal_Sup_R	Supp_Motor_Area_R	FA
Frontal_Sup_Orb_L	Supp_Motor_Area_R	FA
Supp_Motor_Area_R	Olfactory_R	FA
Frontal_Sup_R	Frontal_Sup_Medial_L	FA
Frontal_Inf_Oper_L	Frontal_Sup_Medial_L	FA
Precentral_R	Frontal_Med_Orb_R	FA
Frontal_Inf_Orb_L	Cingulum_Ant_L	FA
Rolandic_Oper_L	Cingulum_Ant_L	FA
Rectus_L	Cingulum_Ant_L	FA
Frontal_Inf_Orb_L	Hippocampus_L	FA
Supp_Motor_Area_R	Hippocampus_R	FA
Frontal_Med_Orb_R	Amygdala_L	FA
Hippocampus_R	Cuneus_R	FA
Hippocampus_L	Occipital_Sup_L	FA

Region1	Region2	Weight
Cuneus_R	Occipital_Sup_L	FA
Insula_L	Occipital_Sup_R	FA
Hippocampus_R	Occipital_Sup_R	FA
Insula_R	Postcentral_R	FA
Frontal_Inf_Orb_L	Parietal_Sup_L	FA
Olfactory_L	Parietal_Sup_L	FA
Precentral_R	Angular_R	FA
Frontal_Mid_R	Angular_R	FA
Occipital_Inf_L	Precuneus_L	FA
Parietal_Sup_L	Precuneus_L	FA
ParaHippocampal_R	Precuneus_R	FA
Amygdala_R	Precuneus_R	FA
Postcentral_R	Precuneus_R	FA
Parietal_Inf_R	Precuneus_R	FA
Frontal_Sup_Orb_L	Paracentral_Lobule_L	FA
Frontal_Mid_Orb_L	Paracentral_Lobule_L	FA
Frontal_Inf_Orb_L	Paracentral_Lobule_L	FA
Supp_Motor_Area_R	Paracentral_Lobule_L	FA
Frontal_Sup_Medial_R	Paracentral_Lobule_L	FA
Amygdala_L	Paracentral_Lobule_L	FA
Lingual_R	Paracentral_Lobule_L	FA
Insula_R	Paracentral_Lobule_R	FA
Amygdala_R	Paracentral_Lobule_R	FA
Paracentral_Lobule_L	Paracentral_Lobule_R	FA
Hippocampus_L	Caudate_R	FA
ParaHippocampal_L	Caudate_R	FA
Cuneus_L	Caudate_R	FA
Lingual_L	Caudate_R	FA
Cingulum_Mid_L	Putamen_L	FA
Parietal_Sup_L	Putamen_L	FA
Paracentral_Lobule_L	Putamen_L	FA
Frontal_Sup_Medial_R	Putamen_R	FA
Occipital_Sup_R	Putamen_R	FA
Supp_Motor_Area_R	Pallidum_R	FA

**Region1**	**Region2**	**Weight**
Cingulum_Mid_R	Pallidum_R	FA
Paracentral_Lobule_R	Pallidum_R	FA
Caudate_R	Thalamus_L	FA
Frontal_Inf_Oper_L	Thalamus_R	FA
Angular_L	Thalamus_R	FA
Cingulum_Ant_R	Heschl_L	FA
Caudate_R	Heschl_R	FA
Rectus_L	Temporal_Pole_Sup_L	FA
Cingulum_Ant_L	Temporal_Pole_Sup_L	FA
Paracentral_Lobule_L	Temporal_Pole_Sup_L	FA
Frontal_Inf_Oper_R	Temporal_Mid_R	FA
Lingual_L	Temporal_Mid_R	FA
Hippocampus_L	Temporal_Pole_Mid_L	FA
Parietal_Sup_L	Temporal_Pole_Mid_L	FA
Supp_Motor_Area_R	Temporal_Pole_Mid_R	FA
Parietal_Sup_L	Temporal_Inf_L	FA
Paracentral_Lobule_L	Temporal_Inf_L	FA
Lingual_L	Temporal_Inf_R	FA
Frontal_Sup_L	Frontal_Inf_Oper_L	FN
Frontal_Mid_Orb_L	Frontal_Inf_Oper_L	FN
Frontal_Mid_R	Frontal_Inf_Oper_R	FN
Frontal_Sup_Orb_L	Supp_Motor_Area_L	FN
Frontal_Sup_Medial_R	Frontal_Med_Orb_R	FN
Frontal_Sup_Medial_L	Rectus_L	FN
Supp_Motor_Area_R	Rectus_R	FN
Frontal_Inf_Tri_R	Insula_R	FN
Precentral_L	Cingulum_Mid_L	FN
Frontal_Mid_L	Cingulum_Mid_L	FN
Rolandic_Oper_L	Amygdala_L	FN
Frontal_Med_Orb_R	Amygdala_L	FN
Cingulum_Ant_R	Amygdala_L	FN
Precentral_R	Calcarine_R	FN
Calcarine_L	Cuneus_L	FN
Hippocampus_R	Cuneus_R	FN

Region1	Region2	Weight
Cuneus_L	Lingual_L	FN
Frontal_Mid_R	Lingual_R	FN
Frontal_Mid_Orb_R	Lingual_R	FN
Frontal_Inf_Orb_R	Lingual_R	FN
Frontal_Inf_Orb_R	Occipital_Sup_R	FN
Cuneus_R	Occipital_Inf_L	FN
Frontal_Sup_Orb_L	Postcentral_L	FN
Insula_R	Postcentral_R	FN
Occipital_Inf_L	Parietal_Sup_L	FN
Frontal_Sup_Orb_L	Parietal_Inf_L	FN
Postcentral_L	Parietal_Inf_L	FN
Cingulum_Ant_R	Angular_L	FN
Cingulum_Post_R	Precuneus_L	FN
Calcarine_L	Precuneus_L	FN
Occipital_Inf_L	Precuneus_L	FN
Lingual_L	Precuneus_R	FN
Lingual_R	Precuneus_R	FN
Frontal_Med_Orb_R	Paracentral_Lobule_R	FN
Supp_Motor_Area_R	Caudate_R	FN
Occipital_Sup_L	Caudate_R	FN
Frontal_Sup_Orb_L	Putamen_L	FN
Precentral_R	Putamen_R	FN
Lingual_R	Putamen_R	FN
Postcentral_R	Putamen_R	FN
Precuneus_R	Putamen_R	FN
Frontal_Sup_Orb_L	Pallidum_L	FN
Supp_Motor_Area_L	Pallidum_L	FN
Cingulum_Mid_L	Pallidum_L	FN
Paracentral_Lobule_L	Pallidum_L	FN
Precentral_R	Pallidum_R	FN
Frontal_Inf_Tri_R	Pallidum_R	FN
Postcentral_R	Pallidum_R	FN
Cingulum_Mid_L	Thalamus_L	FN
Fusiform_L	Thalamus_L	FN

Region1	Region2	Weight
Caudate_L	Thalamus_L	FN
Rolandic_Oper_R	Thalamus_R	FN
Postcentral_R	Thalamus_R	FN
Supp_Motor_Area_L	Temporal_Pole_Sup_L	FN
Temporal_Sup_L	Temporal_Mid_L	FN
Supp_Motor_Area_L	Temporal_Pole_Mid_L	FN
SupraMarginal_L	Temporal_Pole_Mid_L	FN
Precuneus_R	Temporal_Pole_Mid_R	FN
Frontal_Sup_L	Temporal_Inf_L	FN

Table S3. Correlations between cognitive function and changes in brain communication.

Cognition	Weight	Region1	Region2	Report
MMSE	FA_AAL	Frontal_Inf_Orb_L	Hippocampus_L	r=-0.4,P=0.03,Pfdr=0.28
MMSE	FA_AAL	Hippocampus_R	Occipital_Sup_R	r=-0.51,P=0.01,Pfdr=0.14
MMSE	FA_AAL	Frontal_Inf_Orb_L	Parietal_Sup_L	r=-0.49,P=0.01,Pfdr=0.14
MMSE	FA_AAL	Precentral_R	Angular_R	r=-0.44,P=0.02,Pfdr=0.26
MMSE	FA_AAL	Lingual_R	Paracentral_Lobule_L	r=-0.42,P=0.02,Pfdr=0.28
MMSE	FA_AAL	Hippocampus_L	Caudate_R	r=0.4,P=0.04,Pfdr=0.28
MMSE	FA_AAL	Parietal_Sup_L	Putamen_L	r=-0.51,P=0.01,Pfdr=0.14
MMSE	FA_AAL	Frontal_Sup_Medial_R	Putamen_R	r=-0.52,P<0.01,Pfdr=0.14
MMSE	FA_AAL	Occipital_Sup_R	Putamen_R	r=-0.39,P=0.04,Pfdr=0.29
MMSE	FA_AAL	Supp_Motor_Area_R	Temporal_Pole_Mid_R	r=-0.41,P=0.03,Pfdr=0.28
MoCA	FA_AAL	Precentral_R	Angular_R	r=-0.43,P=0.02,Pfdr=0.69
MoCA	FA_AAL	Frontal_Sup_Medial_R	Putamen_R	r=-0.39,P=0.04,Pfdr=0.69
HAMD	FA_AAL	Frontal_Inf_Orb_L	Cingulum_Ant_L	r=-0.44,P=0.02,Pfdr=0.57
HAMD	FA_AAL	Precentral_R	Angular_R	r=0.4,P=0.03,Pfdr=0.57

Cognition	Weight	Region1	Region2	Report
HAMD	FA_AAL	Frontal_Sup_Medial_R	Paracentral_Lobule_L	r=-0.38,P=0.04,Pfdr=0.57
HAMD	FA_AAL	Lingual_L	Caudate_R	r=-0.46,P=0.01,Pfdr=0.57
HAMA	FA_AAL	Lingual_L	Caudate_R	r=-0.4,P=0.04,Pfdr=0.94
HAMA	FA_AAL	Lingual_L	Temporal_Inf_R	r=-0.41,P=0.03,Pfdr=0.94
EM	FA_AAL	Olfactory_L	Parietal_Sup_L	r=0.38,P=0.05,Pfdr=0.95
ISP	FA_AAL	Olfactory_L	Parietal_Sup_L	r=0.38,P=0.05,Pfdr=0.9
ISP	FA_AAL	Supp_Motor_Area_R	Pallidum_R	r=-0.56, P<0.01,Pfdr=0.13
LF	FA_AAL	Occipital_Inf_L	Precuneus_L	r=-0.38,P=0.05,Pfdr=0.67
LF	FA_AAL	Amygdala_R	Precuneus_R	r=-0.4,P=0.04,Pfdr=0.67
LF	FA_AAL	Parietal_Inf_R	Precuneus_R	r=-0.39,P=0.04,Pfdr=0.67
LF	FA_AAL	Paracentral_Lobule_L	Temporal_Pole_Sup_L	r=0.38,P=0.05,Pfdr=0.67
LF	FA_AAL	Parietal_Sup_L	Temporal_Pole_Mid_L	r=0.42,P=0.03,Pfdr=0.67
EF	FA_AAL	Olfactory_L	Parietal_Sup_L	r=0.43,P=0.02,Pfdr=0.41
EF	FA_AAL	Amygdala_R	Paracentral_Lobule_R	r=0.41,P=0.03,Pfdr=0.43
EF	FA_AAL	Paracentral_Lobule_L	Paracentral_Lobule_R	r=-0.62, P<0.01,Pfdr=0.03
EF	FA_AAL	Hippocampus_L	Caudate_R	r=-0.48,P=0.01,Pfdr=0.21
EF	FA_AAL	Rectus_L	Temporal_Pole_Sup_L	r=-0.57, P<0.01,Pfdr=0.06
VS	FA_AAL	Precentral_R	Supp_Motor_Area_R	r=0.44,P=0.02,Pfdr=0.44
VS	FA_AAL	Precentral_R	Angular_R	r=-0.42,P=0.03,Pfdr=0.44
VS	FA_AAL	Postcentral_R	Precuneus_R	r=0.38,P=0.04,Pfdr=0.44
VS	FA_AAL	Hippocampus_L	Caudate_R	r=0.4,P=0.04,Pfdr=0.44
VS	FA_AAL	Angular_L	Thalamus_R	r=0.6, P<0.01,Pfdr=0.05
VS	FA_AAL	Cingulum_Ant_L	Temporal_Pole_Sup_L	r=0.38,P=0.04,Pfdr=0.44
MMSE	FN_AAL	Frontal_Sup_Orb_L	Supp_Motor_Area_L	r=-0.6, P<0.01,Pfdr=0.03
MMSE	FN_AAL	Frontal_Sup_Medial_L	Rectus_L	r=-0.51,P=0.01,Pfdr=0.06
MMSE	FN_AAL	Postcentral_L	Parietal_Inf_L	r=0.4,P=0.03,Pfdr=0.2
MMSE	FN_AAL	Frontal_Med_Orb_R	Paracentral_Lobule_R	r=-0.41,P=0.03,Pfdr=0.2
MMSE	FN_AAL	Occipital_Sup_L	Caudate_R	r=0.57, P<0.01,Pfdr=0.03
MMSE	FN_AAL	Frontal_Sup_Orb_L	Putamen_L	r=-0.57, P<0.01Pfdr=0.03
MMSE	FN_AAL	Precentral_R	Putamen_R	r=-0.41,P=0.03,Pfdr=0.2
MMSE	FN_AAL	Frontal_Sup_Orb_L	Pallidum_L	r=-0.51,P=0.01,Pfdr=0.06
MMSE	FN_AAL	Supp_Motor_Area_L	Pallidum_L	r=-0.49,P=0.01,Pfdr=0.08
MMSE	FN_AAL	Postcentral_R	Pallidum_R	r=0.38,P=0.05,Pfdr=0.24
MMSE	FN_AAL	Caudate_L	Thalamus_L	r=-0.38,P=0.05,Pfdr=0.24

Cognition	Weight	Region1	Region2	Report
MMSE	FN_AAL	Frontal_Sup_L	Temporal_Inf_L	r=-0.48,P=0.01,Pfdr=0.08
HAMD	FN_AAL	Calcarine_L	Precuneus_L	r=0.38,P=0.05,Pfdr=0.62
HAMD	FN_AAL	Occipital_Inf_L	Precuneus_L	r=0.41,P=0.03,Pfdr=0.62
HAMD	FN_AAL	Frontal_Inf_Tri_R	Pallidum_R	r=0.38,P=0.05,Pfdr=0.62
HAMA	FN_AAL	Occipital_Inf_L	Precuneus_L	r=0.39,P=0.04,Pfdr=0.82
HAMA	FN_AAL	Supp_Motor_Area_L	Pallidum_L	r=0.46,P=0.01,Pfdr=0.82
HAMA	FN_AAL	Cingulum_Mid_L	Thalamus_L	r=0.38,P=0.05,Pfdr=0.82
EM	FN_AAL	Precuneus_R	Putamen_R	r=-0.38,P=0.05,Pfdr=0.99
ISP	FN_AAL	Cingulum_Mid_L	Thalamus_L	r=0.38,P=0.05,Pfdr=0.98
LF	FN_AAL	Occipital_Inf_L	Precuneus_L	r=-0.4,P=0.04,Pfdr=0.98
LF	FN_AAL	Precuneus_R	Putamen_R	r=-0.39,P=0.04,Pfdr=0.98
EF	FN_AAL	Frontal_Sup_L	Frontal_Inf_Oper_L	r=0.38,P=0.04,Pfdr=0.62
EF	FN_AAL	Frontal_Sup_Orb_L	Postcentral_L	r=0.51,P=0.01,Pfdr=0.33
EF	FN_AAL	Frontal_Sup_Orb_L	Parietal_Inf_L	r=0.39,P=0.04,Pfdr=0.62
VS	FN_AAL	Frontal_Inf_Tri_R	Insula_R	r=-0.4,P=0.04,Pfdr=0.76
VS	FN_AAL	Postcentral_R	Thalamus_R	r=-0.39,P=0.04,Pfdr=0.76

Table S4. Importance of each variable in the random forest models.

Weight	Cognition	Varible	Importance
FA_AAL	MMSE	cognition_baseline	3.51235413
FA_AAL	MMSE	sex	0.18464778
FA_AAL	MMSE	education	0.43837012
FA_AAL	MMSE	age	4.38423933
FA_AAL	MMSE	edge_15_37	0.35166559
FA_AAL	MMSE	edge_38_50	0.34937048
FA_AAL	MMSE	edge_15_59	0.35622942
FA_AAL	MMSE	edge_2_66	0.45042213
FA_AAL	MMSE	edge_48_69	0.65753027
FA_AAL	MMSE	edge_37_72	0.35143943
FA_AAL	MMSE	edge_59_73	0.44432574
FA_AAL	MMSE	edge_24_74	0.91048067
FA_AAL	MMSE	edge_50_74	0.36017571
FA_AAL	MMSE	edge_20_88	0.65245855

Weight	Cognition	Varible	Importance
FA_AAL	MMSE	edge_15_31	0.52531405
FA_AAL	MMSE	edge_24_69	0.29871546
FA_AAL	MMSE	edge_47_72	0.4557423
FA_AAL	MMSE	edge_47_90	0.55292489
FA_AAL	MMSE	edge_21_59	0.76427608
FA_AAL	MMSE	edge_20_76	0.37464713
FA_AAL	MMSE	edge_53_67	1.09133708
FA_AAL	MMSE	edge_42_68	1.55163429
FA_AAL	MMSE	edge_62_68	0.50847434
FA_AAL	MMSE	edge_69_83	0.50024894
FA_AAL	MMSE	edge_59_87	0.29655918
FA_AAL	MMSE	edge_42_70	0.19045247
FA_AAL	MMSE	edge_69_70	0.99236117
FA_AAL	MMSE	edge_27_83	0.40876873
FA_AAL	MMSE	edge_2_20	0.96772566
FA_AAL	MMSE	edge_58_68	0.75447475
FA_AAL	MMSE	edge_65_78	0.35410194
FA_AAL	MMSE	edge_31_83	1.14349565
FA_AAL	MoCA	cognition_baseline	4.05301675
FA_AAL	MoCA	sex	0.02450611
FA_AAL	MoCA	education	0.18450532
FA_AAL	MoCA	age	0.4635393
FA_AAL	MoCA	edge_15_37	0.34401639
FA_AAL	MoCA	edge_38_50	0.81173469
FA_AAL	MoCA	edge_15_59	0.63607263
FA_AAL	MoCA	edge_2_66	0.85733372
FA_AAL	MoCA	edge_48_69	1.34475489
FA_AAL	MoCA	edge_37_72	0.19058806
FA_AAL	MoCA	edge_59_73	1.04050011
FA_AAL	MoCA	edge_24_74	0.82522963
FA_AAL	MoCA	edge_50_74	2.02607766
FA_AAL	MoCA	edge_20_88	1.30874928
FA_AAL	MoCA	edge_15_31	0.29988195
FA_AAL	MoCA	edge_24_69	0.42789788
FA_AAL	MoCA	edge_47_72	1.08113021
FA_AAL	MoCA	edge_47_90	0.16573779
FA_AAL	MoCA	edge_21_59	0.89021077
FA_AAL	MoCA	edge_20_76	0.22015979

Weight	Cognition	Varible	Importance
FA_AAL	MoCA	edge_53_67	0.87563662
FA_AAL	MoCA	edge_42_68	1.32773088
FA_AAL	MoCA	edge_62_68	1.12443376
FA_AAL	MoCA	edge_69_83	0.45672313
FA_AAL	MoCA	edge_59_87	0.65386153
FA_AAL	MoCA	edge_42_70	0.62581639
FA_AAL	MoCA	edge_69_70	0.519283
FA_AAL	MoCA	edge_27_83	0.29856617
FA_AAL	MoCA	edge_2_20	0.29147557
FA_AAL	MoCA	edge_58_68	0.56105086
FA_AAL	MoCA	edge_65_78	0.53079547
FA_AAL	MoCA	edge_31_83	0.2543207
FA_AAL	HAMD	cognition_baseline	0.98976602
FA_AAL	HAMD	sex	0.09442534
FA_AAL	HAMD	education	0.58857498
FA_AAL	HAMD	age	0.74264448
FA_AAL	HAMD	edge_15_37	0.60461315
FA_AAL	HAMD	edge_38_50	0.53627776
FA_AAL	HAMD	edge_15_59	0.81136141
FA_AAL	HAMD	edge_2_66	0.9097794
FA_AAL	HAMD	edge_48_69	0.53350356
FA_AAL	HAMD	edge_37_72	0.93800464
FA_AAL	HAMD	edge_59_73	0.78203556
FA_AAL	HAMD	edge_24_74	0.59340779
FA_AAL	HAMD	edge_50_74	0.84814098
FA_AAL	HAMD	edge_20_88	0.63578931
FA_AAL	HAMD	edge_15_31	0.81835074
FA_AAL	HAMD	edge_24_69	0.26893706
FA_AAL	HAMD	edge_47_72	0.53607347
FA_AAL	HAMD	edge_47_90	0.44029408
FA_AAL	HAMD	edge_21_59	0.67671275
FA_AAL	HAMD	edge_20_76	0.75369859
FA_AAL	HAMD	edge_53_67	0.7649485
FA_AAL	HAMD	edge_42_68	0.93686941
FA_AAL	HAMD	edge_62_68	0.47890233
FA_AAL	HAMD	edge_69_83	1.02492694
FA_AAL	HAMD	edge_59_87	0.88708179
FA_AAL	HAMD	edge_42_70	0.55961122

Weight	Cognition	Varible	Importance
FA_AAL	HAMD	edge_69_70	1.02849951
FA_AAL	HAMD	edge_27_83	0.57607639
FA_AAL	HAMD	edge_2_20	0.47416864
FA_AAL	HAMD	edge_58_68	0.53516076
FA_AAL	HAMD	edge_65_78	1.24928613
FA_AAL	HAMD	edge_31_83	0.60612766
FA_AAL	HAMA	cognition_baseline	10.0687857
FA_AAL	HAMA	sex	0.0261382
FA_AAL	HAMA	education	0.05695049
FA_AAL	HAMA	age	0.321813
FA_AAL	HAMA	edge_15_37	0.67125034
FA_AAL	HAMA	edge_38_50	0.54257792
FA_AAL	HAMA	edge_15_59	0.92067359
FA_AAL	HAMA	edge_2_66	0.66852137
FA_AAL	HAMA	edge_48_69	0.30613847
FA_AAL	HAMA	edge_37_72	0.58058887
FA_AAL	HAMA	edge_59_73	0.17498562
FA_AAL	HAMA	edge_24_74	0.75229276
FA_AAL	HAMA	edge_50_74	0.53621589
FA_AAL	HAMA	edge_20_88	0.1473736
FA_AAL	HAMA	edge_15_31	0.2440472
FA_AAL	HAMA	edge_24_69	0.3446487
FA_AAL	HAMA	edge_47_72	0.25051223
FA_AAL	HAMA	edge_47_90	0.18285195
FA_AAL	HAMA	edge_21_59	1.1200126
FA_AAL	HAMA	edge_20_76	0.42202608
FA_AAL	HAMA	edge_53_67	0.29716878
FA_AAL	HAMA	edge_42_68	0.55027734
FA_AAL	HAMA	edge_62_68	0.12469432
FA_AAL	HAMA	edge_69_83	1.7138562
FA_AAL	HAMA	edge_59_87	0.34201111
FA_AAL	HAMA	edge_42_70	1.44905528
FA_AAL	HAMA	edge_69_70	0.26330037
FA_AAL	HAMA	edge_27_83	0.35831152
FA_AAL	HAMA	edge_2_20	0.12571637
FA_AAL	HAMA	edge_58_68	0.18154157
FA_AAL	HAMA	edge_65_78	1.96408564
FA_AAL	HAMA	edge_31_83	0.2229468

Weight	Cognition	Varible	Importance
FA_AAL	EM	cognition_baseline	14.3213738
FA_AAL	EM	sex	0.00228272
FA_AAL	EM	education	0.12968246
FA_AAL	EM	age	0.25270929
FA_AAL	EM	edge_15_37	0.6240654
FA_AAL	EM	edge_38_50	1.68241625
FA_AAL	EM	edge_15_59	0.28797292
FA_AAL	EM	edge_2_66	0.06802633
FA_AAL	EM	edge_48_69	0.48221063
FA_AAL	EM	edge_37_72	0.16616976
FA_AAL	EM	edge_59_73	0.23403407
FA_AAL	EM	edge_24_74	0.81552448
FA_AAL	EM	edge_50_74	0.31610342
FA_AAL	EM	edge_20_88	0.32832256
FA_AAL	EM	edge_15_31	0.95009346
FA_AAL	EM	edge_24_69	0.44261002
FA_AAL	EM	edge_47_72	0.12633056
FA_AAL	EM	edge_47_90	0.14476107
FA_AAL	EM	edge_21_59	0.06011696
FA_AAL	EM	edge_20_76	0.12005686
FA_AAL	EM	edge_53_67	0.26091107
FA_AAL	EM	edge_42_68	0.2836649
FA_AAL	EM	edge_62_68	0.68037469
FA_AAL	EM	edge_69_83	0.29202713
FA_AAL	EM	edge_59_87	0.23942813
FA_AAL	EM	edge_42_70	0.79063388
FA_AAL	EM	edge_69_70	0.3480639
FA_AAL	EM	edge_27_83	0.12582805
FA_AAL	EM	edge_2_20	0.35968784
FA_AAL	EM	edge_58_68	0.18102561
FA_AAL	EM	edge_65_78	0.1285716
FA_AAL	EM	edge_31_83	0.11567766
FA_AAL	ISP	cognition_baseline	14.1527911
FA_AAL	ISP	sex	0.0224298
FA_AAL	ISP	education	0.18621945
FA_AAL	ISP	age	0.54819645
FA_AAL	ISP	edge_15_37	1.10880553
FA_AAL	ISP	edge_38_50	0.2364935

Weight	Cognition	Varible	Importance
FA_AAL	ISP	edge_15_59	0.2751174
FA_AAL	ISP	edge_2_66	0.09119451
FA_AAL	ISP	edge_48_69	0.0981766
FA_AAL	ISP	edge_37_72	0.09211358
FA_AAL	ISP	edge_59_73	0.16355805
FA_AAL	ISP	edge_24_74	0.22318092
FA_AAL	ISP	edge_50_74	1.86659248
FA_AAL	ISP	edge_20_88	0.09272567
FA_AAL	ISP	edge_15_31	0.25111675
FA_AAL	ISP	edge_24_69	0.21335731
FA_AAL	ISP	edge_47_72	0.28945225
FA_AAL	ISP	edge_47_90	0.21031614
FA_AAL	ISP	edge_21_59	0.3447769
FA_AAL	ISP	edge_20_76	0.26652685
FA_AAL	ISP	edge_53_67	0.165328
FA_AAL	ISP	edge_42_68	0.10814453
FA_AAL	ISP	edge_62_68	0.21688185
FA_AAL	ISP	edge_69_83	0.62147344
FA_AAL	ISP	edge_59_87	0.35382538
FA_AAL	ISP	edge_42_70	0.38232408
FA_AAL	ISP	edge_69_70	0.39568693
FA_AAL	ISP	edge_27_83	0.14241539
FA_AAL	ISP	edge_2_20	0.14099167
FA_AAL	ISP	edge_58_68	0.11746049
FA_AAL	ISP	edge_65_78	1.85455203
FA_AAL	ISP	edge_31_83	0.14895397
FA_AAL	LF	cognition_baseline	18.9268181
FA_AAL	LF	sex	0.01287603
FA_AAL	LF	education	0.03160512
FA_AAL	LF	age	0.54151875
FA_AAL	LF	edge_15_37	0.21305418
FA_AAL	LF	edge_38_50	0.09682081
FA_AAL	LF	edge_15_59	0.28062937
FA_AAL	LF	edge_2_66	0.32887255
FA_AAL	LF	edge_48_69	0.07541173
FA_AAL	LF	edge_37_72	0.05181738
FA_AAL	LF	edge_59_73	0.35709324
FA_AAL	LF	edge_24_74	0.12322971

Weight	Cognition	Varible	Importance
FA_AAL	LF	edge_50_74	0.66840483
FA_AAL	LF	edge_20_88	0.12649265
FA_AAL	LF	edge_15_31	0.18781394
FA_AAL	LF	edge_24_69	0.03896277
FA_AAL	LF	edge_47_72	0.22223211
FA_AAL	LF	edge_47_90	0.19358326
FA_AAL	LF	edge_21_59	0.26880299
FA_AAL	LF	edge_20_76	0.1223154
FA_AAL	LF	edge_53_67	0.06822922
FA_AAL	LF	edge_42_68	0.10484078
FA_AAL	LF	edge_62_68	0.05302307
FA_AAL	LF	edge_69_83	0.18304442
FA_AAL	LF	edge_59_87	0.07063242
FA_AAL	LF	edge_42_70	0.71279109
FA_AAL	LF	edge_69_70	0.20303376
FA_AAL	LF	edge_27_83	0.06823377
FA_AAL	LF	edge_2_20	0.10845861
FA_AAL	LF	edge_58_68	0.15716206
FA_AAL	LF	edge_65_78	0.4881107
FA_AAL	LF	edge_31_83	0.31729135
FA_AAL	EF	cognition_baseline	11.6089463
FA_AAL	EF	sex	0.22652851
FA_AAL	EF	education	0.18779895
FA_AAL	EF	age	0.3997498
FA_AAL	EF	edge_15_37	0.22405847
FA_AAL	EF	edge_38_50	2.17279852
FA_AAL	EF	edge_15_59	0.34532551
FA_AAL	EF	edge_2_66	0.55092077
FA_AAL	EF	edge_48_69	0.88268899
FA_AAL	EF	edge_37_72	0.0958613
FA_AAL	EF	edge_59_73	0.9950534
FA_AAL	EF	edge_24_74	0.58848546
FA_AAL	EF	edge_50_74	1.28032309
FA_AAL	EF	edge_20_88	1.12750164
FA_AAL	EF	edge_15_31	0.40130355
FA_AAL	EF	edge_24_69	0.11515167
FA_AAL	EF	edge_47_72	0.19714339
FA_AAL	EF	edge_47_90	0.19881795

Weight	Cognition	Varible	Importance
FA_AAL	EF	edge_21_59	0.69261357
FA_AAL	EF	edge_20_76	0.37689423
FA_AAL	EF	edge_53_67	0.67940751
FA_AAL	EF	edge_42_68	0.08179625
FA_AAL	EF	edge_62_68	0.37418435
FA_AAL	EF	edge_69_83	0.23065348
FA_AAL	EF	edge_59_87	0.10891034
FA_AAL	EF	edge_42_70	0.45423197
FA_AAL	EF	edge_69_70	0.16804005
FA_AAL	EF	edge_27_83	0.18095958
FA_AAL	EF	edge_2_20	0.28653358
FA_AAL	EF	edge_58_68	0.1472812
FA_AAL	EF	edge_65_78	0.25195247
FA_AAL	EF	edge_31_83	0.12125693
FA_AAL	VS	cognition_baseline	13.9838456
FA_AAL	VS	sex	0.01188449
FA_AAL	VS	education	0.29344788
FA_AAL	VS	age	0.53417165
FA_AAL	VS	edge_15_37	0.31417731
FA_AAL	VS	edge_38_50	0.49713208
FA_AAL	VS	edge_15_59	0.11009177
FA_AAL	VS	edge_2_66	0.1770223
FA_AAL	VS	edge_48_69	0.19439276
FA_AAL	VS	edge_37_72	0.06739763
FA_AAL	VS	edge_59_73	0.23168546
FA_AAL	VS	edge_24_74	0.42961261
FA_AAL	VS	edge_50_74	0.12282343
FA_AAL	VS	edge_20_88	0.57715156
FA_AAL	VS	edge_15_31	0.0714149
FA_AAL	VS	edge_24_69	0.09540177
FA_AAL	VS	edge_47_72	0.20786111
FA_AAL	VS	edge_47_90	0.69565687
FA_AAL	VS	edge_21_59	0.16993572
FA_AAL	VS	edge_20_76	0.05370548
FA_AAL	VS	edge_53_67	1.02932916
FA_AAL	VS	edge_42_68	0.15942553
FA_AAL	VS	edge_62_68	0.17414001
FA_AAL	VS	edge_69_83	0.20527139

Weight	Cognition	Varible	Importance
FA_AAL	VS	edge_59_87	0.28933438
FA_AAL	VS	edge_42_70	0.08788335
FA_AAL	VS	edge_69_70	2.20266578
FA_AAL	VS	edge_27_83	0.1300684
FA_AAL	VS	edge_2_20	0.57420563
FA_AAL	VS	edge_58_68	0.10509278
FA_AAL	VS	edge_65_78	0.12012837
FA_AAL	VS	edge_31_83	0.20526534
FN_AAL	MMSE	cognition_baseline	5.39736304
FN_AAL	MMSE	sex	0.02273188
FN_AAL	MMSE	education	0.34374865
FN_AAL	MMSE	age	5.53957503
FN_AAL	MMSE	edge_5_19	0.22986214
FN_AAL	MMSE	edge_23_27	0.24109981
FN_AAL	MMSE	edge_57_61	1.43486684
FN_AAL	MMSE	edge_26_70	0.79697803
FN_AAL	MMSE	edge_49_72	0.27828408
FN_AAL	MMSE	edge_5_73	0.22161218
FN_AAL	MMSE	edge_2_74	3.97209951
FN_AAL	MMSE	edge_5_75	0.4060268
FN_AAL	MMSE	edge_19_75	0.6739438
FN_AAL	MMSE	edge_58_76	0.49495168
FN_AAL	MMSE	edge_71_77	0.14812605
FN_AAL	MMSE	edge_3_89	0.40591572
FN_AAL	MMSE	edge_43_67	0.23939182
FN_AAL	MMSE	edge_53_67	0.31111572
FN_AAL	MMSE	edge_14_76	0.41925345
FN_AAL	MMSE	edge_33_77	0.210984
FN_AAL	MMSE	edge_68_74	1.18425913
FN_AAL	MMSE	edge_3_11	0.51764739
FN_AAL	MMSE	edge_5_57	0.17494166
FN_AAL	MMSE	edge_5_61	0.33825791
FN_AAL	MMSE	edge_14_30	0.30128782
FN_AAL	MMSE	edge_58_78	0.66566788
FN_AAL	MoCA	cognition_baseline	9.11821692
FN_AAL	MoCA	sex	0.03772953
FN_AAL	MoCA	education	0.22890334
FN_AAL	MoCA	age	0.41629525

Weight	Cognition	Varible	Importance
FN_AAL	MoCA	edge_5_19	0.31041676
FN_AAL	MoCA	edge_23_27	2.96455183
FN_AAL	MoCA	edge_57_61	0.28097371
FN_AAL	MoCA	edge_26_70	0.50706642
FN_AAL	MoCA	edge_49_72	0.62904998
FN_AAL	MoCA	edge_5_73	0.20822426
FN_AAL	MoCA	edge_2_74	0.50457984
FN_AAL	MoCA	edge_5_75	0.18494277
FN_AAL	MoCA	edge_19_75	0.79318911
FN_AAL	MoCA	edge_58_76	0.57550171
FN_AAL	MoCA	edge_71_77	0.37687034
FN_AAL	MoCA	edge_3_89	0.95313262
FN_AAL	MoCA	edge_43_67	0.87751422
FN_AAL	MoCA	edge_53_67	1.27882787
FN_AAL	MoCA	edge_14_76	1.27683754
FN_AAL	MoCA	edge_33_77	0.86339446
FN_AAL	MoCA	edge_68_74	0.33931656
FN_AAL	MoCA	edge_3_11	0.24355212
FN_AAL	MoCA	edge_5_57	0.44675505
FN_AAL	MoCA	edge_5_61	0.27752066
FN_AAL	MoCA	edge_14_30	0.58666343
FN_AAL	MoCA	edge_58_78	0.90538016
FN_AAL	HAMD	cognition_baseline	1.125957
FN_AAL	HAMD	sex	0.15189976
FN_AAL	HAMD	education	0.93255072
FN_AAL	HAMD	age	1.09651146
FN_AAL	HAMD	edge_5_19	1.00320196
FN_AAL	HAMD	edge_23_27	0.75732584
FN_AAL	HAMD	edge_57_61	0.79014623
FN_AAL	HAMD	edge_26_70	1.18995813
FN_AAL	HAMD	edge_49_72	0.73347339
FN_AAL	HAMD	edge_5_73	0.54046704
FN_AAL	HAMD	edge_2_74	1.03893759
FN_AAL	HAMD	edge_5_75	0.89546951
FN_AAL	HAMD	edge_19_75	0.73243411
FN_AAL	HAMD	edge_58_76	0.60429089
FN_AAL	HAMD	edge_71_77	1.53736555
FN_AAL	HAMD	edge_3_89	0.51556835

Weight	Cognition	Varible	Importance
FN_AAL	HAMD	edge_43_67	0.86792335
FN_AAL	HAMD	edge_53_67	0.60426919
FN_AAL	HAMD	edge_14_76	1.04353077
FN_AAL	HAMD	edge_33_77	0.73308426
FN_AAL	HAMD	edge_68_74	0.69829902
FN_AAL	HAMD	edge_3_11	0.86154333
FN_AAL	HAMD	edge_5_57	0.67140462
FN_AAL	HAMD	edge_5_61	0.94505438
FN_AAL	HAMD	edge_14_30	0.87160438
FN_AAL	HAMD	edge_58_78	0.8254113
FN_AAL	HAMA	cognition_baseline	10.1868657
FN_AAL	HAMA	sex	0.0466458
FN_AAL	HAMA	education	0.23810644
FN_AAL	HAMA	age	0.33854801
FN_AAL	HAMA	edge_5_19	0.38794356
FN_AAL	HAMA	edge_23_27	0.60491201
FN_AAL	HAMA	edge_57_61	0.15005062
FN_AAL	HAMA	edge_26_70	1.20379048
FN_AAL	HAMA	edge_49_72	2.15160708
FN_AAL	HAMA	edge_5_73	0.08004943
FN_AAL	HAMA	edge_2_74	0.24163096
FN_AAL	HAMA	edge_5_75	0.25030057
FN_AAL	HAMA	edge_19_75	0.26015329
FN_AAL	HAMA	edge_58_76	0.33565513
FN_AAL	HAMA	edge_71_77	2.44167397
FN_AAL	HAMA	edge_3_89	0.4177089
FN_AAL	HAMA	edge_43_67	0.95588114
FN_AAL	HAMA	edge_53_67	0.08379484
FN_AAL	HAMA	edge_14_76	0.7491567
FN_AAL	HAMA	edge_33_77	1.13874171
FN_AAL	HAMA	edge_68_74	0.82730758
FN_AAL	HAMA	edge_3_11	0.35167016
FN_AAL	HAMA	edge_5_57	0.10369338
FN_AAL	HAMA	edge_5_61	1.80379925
FN_AAL	HAMA	edge_14_30	0.54068402
FN_AAL	HAMA	edge_58_78	0.66700817
FN_AAL	EM	cognition_baseline	16.5486291
FN_AAL	EM	sex	0.00541935

Weight	Cognition	Varible	Importance
FN_AAL	EM	education	0.11954691
FN_AAL	EM	age	0.3181389
FN_AAL	EM	edge_5_19	0.17897201
FN_AAL	EM	edge_23_27	0.25863105
FN_AAL	EM	edge_57_61	0.60968995
FN_AAL	EM	edge_26_70	0.39662348
FN_AAL	EM	edge_49_72	0.59454855
FN_AAL	EM	edge_5_73	0.12028201
FN_AAL	EM	edge_2_74	0.33527308
FN_AAL	EM	edge_5_75	0.29793904
FN_AAL	EM	edge_19_75	0.18452794
FN_AAL	EM	edge_58_76	0.46593494
FN_AAL	EM	edge_71_77	0.2079745
FN_AAL	EM	edge_3_89	0.18638273
FN_AAL	EM	edge_43_67	0.42729933
FN_AAL	EM	edge_53_67	0.45824234
FN_AAL	EM	edge_14_76	0.46367139
FN_AAL	EM	edge_33_77	0.6202888
FN_AAL	EM	edge_68_74	0.87075248
FN_AAL	EM	edge_3_11	0.49385948
FN_AAL	EM	edge_5_57	0.35501707
FN_AAL	EM	edge_5_61	0.48411572
FN_AAL	EM	edge_14_30	0.15724207
FN_AAL	EM	edge_58_78	0.72041808
FN_AAL	ISP	cognition_baseline	14.6692869
FN_AAL	ISP	sex	0.03489899
FN_AAL	ISP	education	0.23186261
FN_AAL	ISP	age	0.69068305
FN_AAL	ISP	edge_5_19	2.21668398
FN_AAL	ISP	edge_23_27	0.14359933
FN_AAL	ISP	edge_57_61	0.14920047
FN_AAL	ISP	edge_26_70	0.3812943
FN_AAL	ISP	edge_49_72	0.38566672
FN_AAL	ISP	edge_5_73	0.28303863
FN_AAL	ISP	edge_2_74	0.62617757
FN_AAL	ISP	edge_5_75	0.1643114
FN_AAL	ISP	edge_19_75	0.41159525
FN_AAL	ISP	edge_58_76	0.64902307

Weight	Cognition	Varible	Importance
FN_AAL	ISP	edge_71_77	1.01451441
FN_AAL	ISP	edge_3_89	0.3543907
FN_AAL	ISP	edge_43_67	0.16437139
FN_AAL	ISP	edge_53_67	0.18208022
FN_AAL	ISP	edge_14_76	0.81542597
FN_AAL	ISP	edge_33_77	0.13146641
FN_AAL	ISP	edge_68_74	0.22871037
FN_AAL	ISP	edge_3_11	0.22146496
FN_AAL	ISP	edge_5_57	0.14021395
FN_AAL	ISP	edge_5_61	0.19229938
FN_AAL	ISP	edge_14_30	0.50214522
FN_AAL	ISP	edge_58_78	0.6430775
FN_AAL	LF	cognition_baseline	18.0239827
FN_AAL	LF	sex	0.01946785
FN_AAL	LF	education	0.13196386
FN_AAL	LF	age	0.7155709
FN_AAL	LF	edge_5_19	0.04618456
FN_AAL	LF	edge_23_27	0.39480123
FN_AAL	LF	edge_57_61	0.30759481
FN_AAL	LF	edge_26_70	0.48586644
FN_AAL	LF	edge_49_72	0.16171411
FN_AAL	LF	edge_5_73	0.05173126
FN_AAL	LF	edge_2_74	0.51067821
FN_AAL	LF	edge_5_75	0.07127003
FN_AAL	LF	edge_19_75	0.22427492
FN_AAL	LF	edge_58_76	0.08296498
FN_AAL	LF	edge_71_77	0.64024616
FN_AAL	LF	edge_3_89	0.24353604
FN_AAL	LF	edge_43_67	0.20534141
FN_AAL	LF	edge_53_67	1.00540746
FN_AAL	LF	edge_14_76	0.19558419
FN_AAL	LF	edge_33_77	0.20604931
FN_AAL	LF	edge_68_74	0.15185678
FN_AAL	LF	edge_3_11	0.6359681
FN_AAL	LF	edge_5_57	0.13619926
FN_AAL	LF	edge_5_61	0.28211763
FN_AAL	LF	edge_14_30	0.18839971
FN_AAL	LF	edge_58_78	0.28260518

Weight	Cognition	Varible	Importance
FN_AAL	EF	cognition_baseline	10.5540192
FN_AAL	EF	sex	0.19000877
FN_AAL	EF	education	0.30419805
FN_AAL	EF	age	0.91598452
FN_AAL	EF	edge_5_19	1.95246791
FN_AAL	EF	edge_23_27	0.56417358
FN_AAL	EF	edge_57_61	0.33319426
FN_AAL	EF	edge_26_70	0.34554399
FN_AAL	EF	edge_49_72	0.32054434
FN_AAL	EF	edge_5_73	0.23541255
FN_AAL	EF	edge_2_74	0.68251534
FN_AAL	EF	edge_5_75	0.24047299
FN_AAL	EF	edge_19_75	0.36435696
FN_AAL	EF	edge_58_76	0.52803372
FN_AAL	EF	edge_71_77	0.51953508
FN_AAL	EF	edge_3_89	0.45717885
FN_AAL	EF	edge_43_67	0.65197793
FN_AAL	EF	edge_53_67	0.96244857
FN_AAL	EF	edge_14_76	0.37852329
FN_AAL	EF	edge_33_77	0.92848874
FN_AAL	EF	edge_68_74	0.17803352
FN_AAL	EF	edge_3_11	0.75842582
FN_AAL	EF	edge_5_57	1.08429964
FN_AAL	EF	edge_5_61	0.68458334
FN_AAL	EF	edge_14_30	0.75477402
FN_AAL	EF	edge_58_78	0.34031905
FN_AAL	VS	cognition_baseline	13.2077921
FN_AAL	VS	sex	0.00456842
FN_AAL	VS	education	0.84825278
FN_AAL	VS	age	0.75291223
FN_AAL	VS	edge_5_19	1.03000741
FN_AAL	VS	edge_23_27	0.10726573
FN_AAL	VS	edge_57_61	1.55311002
FN_AAL	VS	edge_26_70	0.07302387
FN_AAL	VS	edge_49_72	0.13885303
FN_AAL	VS	edge_5_73	0.40328221
FN_AAL	VS	edge_2_74	0.37266375
FN_AAL	VS	edge_5_75	0.23040384

Weight	Cognition	Varible	Importance
FN_AAL	VS	edge_19_75	0.1062968
FN_AAL	VS	edge_58_76	0.72654099
FN_AAL	VS	edge_71_77	0.11040588
FN_AAL	VS	edge_3_89	0.33413971
FN_AAL	VS	edge_43_67	1.54625688
FN_AAL	VS	edge_53_67	0.25222715
FN_AAL	VS	edge_14_76	0.17467974
FN_AAL	VS	edge_33_77	0.92568735
FN_AAL	VS	edge_68_74	0.17994708
FN_AAL	VS	edge_3_11	0.46989529
FN_AAL	VS	edge_5_57	0.08751611
FN_AAL	VS	edge_5_61	0.15943424
FN_AAL	VS	edge_14_30	0.16470962
FN_AAL	VS	edge_58_78	0.79810768

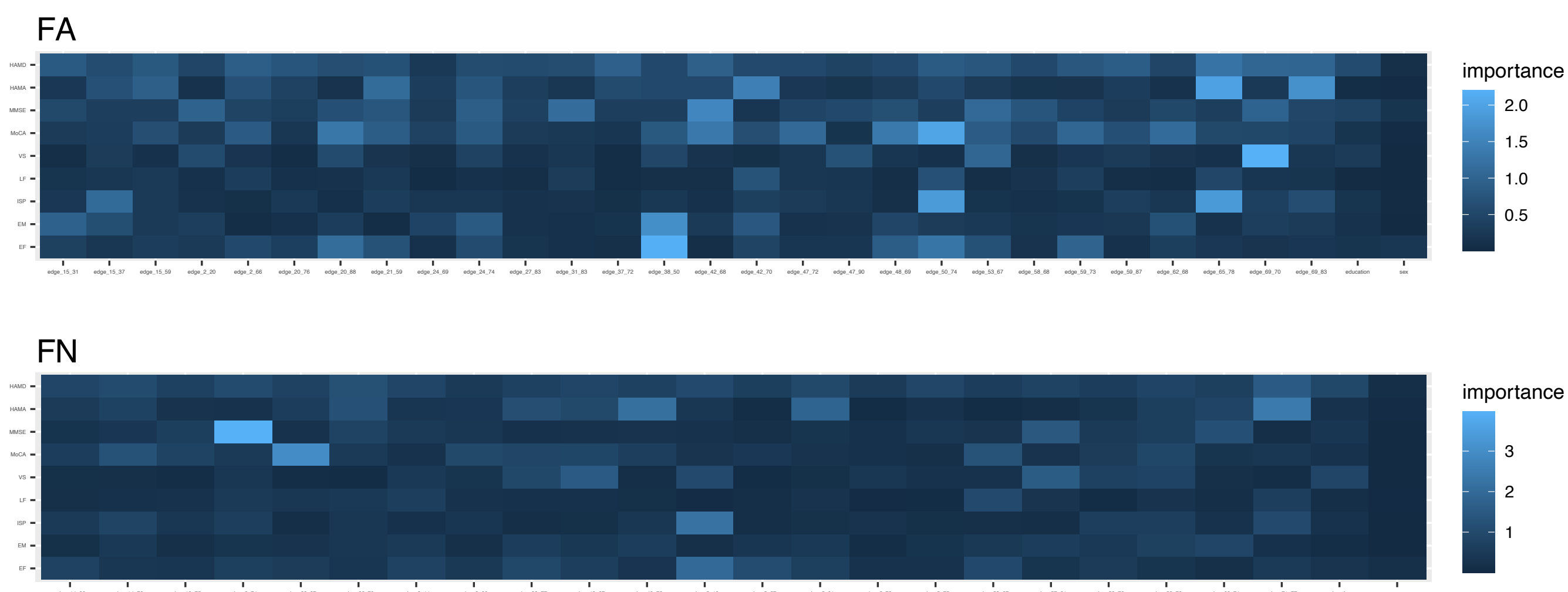

Fig. (S1). Importance of features. Importance of edge-wise communicability features in random forest models. FA, fractional anisotropy; FN, number of fibres.
